# SUMOylation by SUMO2 is implicated in the degradation of misfolded ataxin-7 via RNF4 in SCA7 models

**DOI:** 10.1242/dmm.036145

**Published:** 2019-01-11

**Authors:** Martina Marinello, Andreas Werner, Mariagiovanna Giannone, Khadija Tahiri, Sandro Alves, Christelle Tesson, Wilfred den Dunnen, Jacob-S. Seeler, Alexis Brice, Annie Sittler

**Affiliations:** 1 Sorbonne Universités, UPMC, Univ Paris 06 UMRS 1127, INSERM U 1127, CNRS UMR 7225, ICM (Brain and Spine Institute) Pitié-Salpêtrière Hospital, 75013 Paris, France; 2 Ecole Pratique des Hautes Etudes (EPHE), Paris Sciences et Lettres (PSL) Research University, Neurogenetics Group, 75013 Paris, France; 3 Zentrum für Molekulare Biologie der Universität Heidelberg, DKFZ-ZMBH Alliance, 69120 Heidelberg, Germany; 4 Department of Pathology and Medical Biology, University of Groningen, University Medical Center Groningen, PO Box 30.001, 9700 RB Groningen, The Netherlands; 5 Nuclear Organization and Oncogenesis Unit, INSERM U.993, Department of Cell Biology and Infection, Institut Pasteur, F-75015 Paris, France; 6 AP-HP, Genetic Department, Pitié-Salpêtrière University Hospital, F-75013 Paris, France

**Keywords:** Polyglutamine disease, SCA7, SUMO, Proteasome, RNF4

## Abstract

Perturbation of protein homeostasis and aggregation of misfolded proteins is a major cause of many human diseases. A hallmark of the neurodegenerative disease spinocerebellar ataxia type 7 (SCA7) is the intranuclear accumulation of mutant, misfolded ataxin-7 (polyQ-ATXN7). Here, we show that endogenous ATXN7 is modified by SUMO proteins, thus also suggesting a physiological role for this modification under conditions of proteotoxic stress caused by the accumulation of polyQ-ATXN7. Co-immunoprecipitation experiments, immunofluorescence microscopy and proximity ligation assays confirmed the colocalization and interaction of polyQ-ATXN7 with SUMO2 in cells. Moreover, upon inhibition of the proteasome, both endogenous SUMO2/3 and the RNF4 ubiquitin ligase surround large polyQ-ATXN7 intranuclear inclusions. Overexpression of RNF4 and/or SUMO2 significantly decreased levels of polyQ-ATXN7 and, upon proteasomal inhibition, led to a marked increase in the polyubiquitination of polyQ-ATXN7. This provides a mechanism for the clearance of polyQ-ATXN7 from affected cells that involves the recruitment of RNF4 by SUMO2/3-modified polyQ-ATXN7, thus leading to its ubiquitination and proteasomal degradation. In a SCA7 knock-in mouse model, we similarly observed colocalization of SUMO2/3 with polyQ-ATXN7 inclusions in the cerebellum and retina. Furthermore, we detected accumulation of SUMO2/3 high-molecular-mass species in the cerebellum of SCA7 knock-in mice, compared with their wild-type littermates, and changes in SUMO-related transcripts. Immunohistochemical analysis showed the accumulation of SUMO proteins and RNF4 in the cerebellum of SCA7 patients. Taken together, our results show that the SUMO pathway contributes to the clearance of aggregated ATXN7 and suggest that its deregulation might be associated with SCA7 disease progression.

## Abstract (precis)

**Summary:** In spinocerebellar ataxia type 7, SUMOylation contributes to the clearance of mutant ATXN7. SUMO2-modified ATXN7 interacts with the SUMO-dependent ubiquitin ligase RNF4, which polyubiquitinates ATXN7, promoting its proteasomal degradation.

## INTRODUCTION

Spinocerebellar ataxia type 7 (SCA7) is an inherited neurological disorder characterized by severe loss of neurons in the cerebellum and associated structures, as well as macular degeneration (David et al., 1997; Michalik et al., 2003).

SCA7 is one of nine polyglutamine (polyQ) neurodegenerative disorders, and is caused by CAG/polyQ repeat expansions in the ataxin-7 (*ATXN7*) gene. The polyQ stretch of the ATXN7 protein in healthy individuals consists of 4-35 glutamines, but is expanded to 37->400 glutamines in patients (David et al., 1997; Stevanin et al., 1998; van de Warrenburg et al., 2001).

ATXN7 plays a role in the regulation of transcription, as it is a component of the co-activator multi-protein complex SAGA, involved in histone acetylation (Mohan et al., 2014). Mutant (polyQ) ATXN7 accumulates in the nucleus and forms intranuclear neuronal inclusions due to its polyQ content. These inclusions contain other transcriptional activators or repressors, as well as chaperones, proteasome subunits, promyelocytic leukemia protein (PML) and proteins implicated in post-translational modifications, such as ubiquitin and the small ubiquitin-like modifier (SUMO) proteins (Janer et al., 2010; Takahashi et al., 2003).

Clastosomes were first described by Lafarga et al. (2002) as a subset of PML nuclear bodies that contain components of the ubiquitin-proteasome pathway, including the active 20S core and the 19S regulatory subunits of the proteasome, as well as substrates, such as short-lived transcription factors. Normally scarce, these structures form in response to stimuli that activate proteasome-dependent proteolysis and disappear when proteasome function is inhibited.

SUMOylation is the covalent attachment of a small ubiquitin-related SUMO protein to specific lysine residues in target proteins. This reversible modification is a significant regulatory mechanism of normal cell function, in particular nuclear signaling or transport, transcriptional regulation, protein stability, and DNA replication or repair (Flotho and Melchior, 2013). Mammals express four ∼100-amino-acid SUMO proteins: SUMO1, SUMO2 and SUMO3 [the latter two often referred to as one protein, SUMO2/3 (with 97% identical residues)], and SUMO4. Although SUMO1 and SUMO2/3 use the same enzymatic conjugation pathway, they serve different functions as the various isoforms can be conjugated to different target proteins (Saitoh and Hinchey, 2000; Vertegaal et al., 2006). Furthermore, the ubiquitin-proteasome system acts in a cooperative manner with SUMO2/3 modification (Schimmel et al., 2008), thus linking this specific SUMOylation pathway to protein degradation. SUMO2/3 chains serve as docking sites for RNF4, which earmarks the misfolded proteins for proteasomal degradation by adding a ubiquitin tag. This mechanism was first described in acute promyelocytic leukemia, which can be treated with arsenic, which induces polySUMOylation of PML, recognition by RNF4, subsequent polyubiquitination and proteasomal degradation (Lallemand-Breitenbach et al., 2008; Tatham et al., 2008). The multi-step SUMOylation process requires E1, E2 and E3 activities. In mammals, there is a single E1-activating enzyme, and a single E2-conjugating enzyme (UBC9), but multiple E3 ligases, which provide substrate specificity (Andrews et al., 2005; Kahyo et al., 2001; Pichler et al., 2002). SUMOylation is usually very transient and is reversed by SUMO proteases, e.g. of the SENP family, in such a way that they both cleave the SUMO moiety from its target protein and process SUMO itself during maturation from its precursor (Reverter and Lima, 2006; Shen et al., 2006).

Depending on the modified protein, SUMOylation can act either as a positive or negative regulator in polyQ expansion disease. In a *Drosophila* model of Huntington's disease (HD), genetic reduction of SUMO1 was protective, and SUMOylation decreased the aggregation of the HD exon-1-polyQ protein in a cell model (Steffan et al., 2004). It has been shown that disruption of SUMOylation of the polyQ-androgen receptor enhanced its hormone-dependent transcriptional regulatory activity (Chua et al., 2015). A role of PML as the SUMO E3 ligase for ataxin-1 was uncovered, and it was shown that ataxin-1 with an expansion of 82Q was subjected to SUMO-dependent polyubiquitination by RNF4 and subsequent proteasomal degradation (Guo et al., 2014).

We have shown previously that non-expanded ATXN7 and polyQ-ATXN7 are modified by SUMO on lysine 257, and that SUMOylation affects mutant ATXN7 aggregation (Janer et al., 2010). The aims of the present study were to: (1) further understand the mechanism of mutant ATXN7 SUMOylation and its implication on protein accumulation; (2) elucidate the physiological role of mutant ATXN7 modification by SUMO2; and (3) understand whether a deregulation of the SUMO pathway might contribute to SCA7 pathogenesis.

## RESULTS

### ATXN7 is modified by SUMO2 in cells

**Fig. 1.**
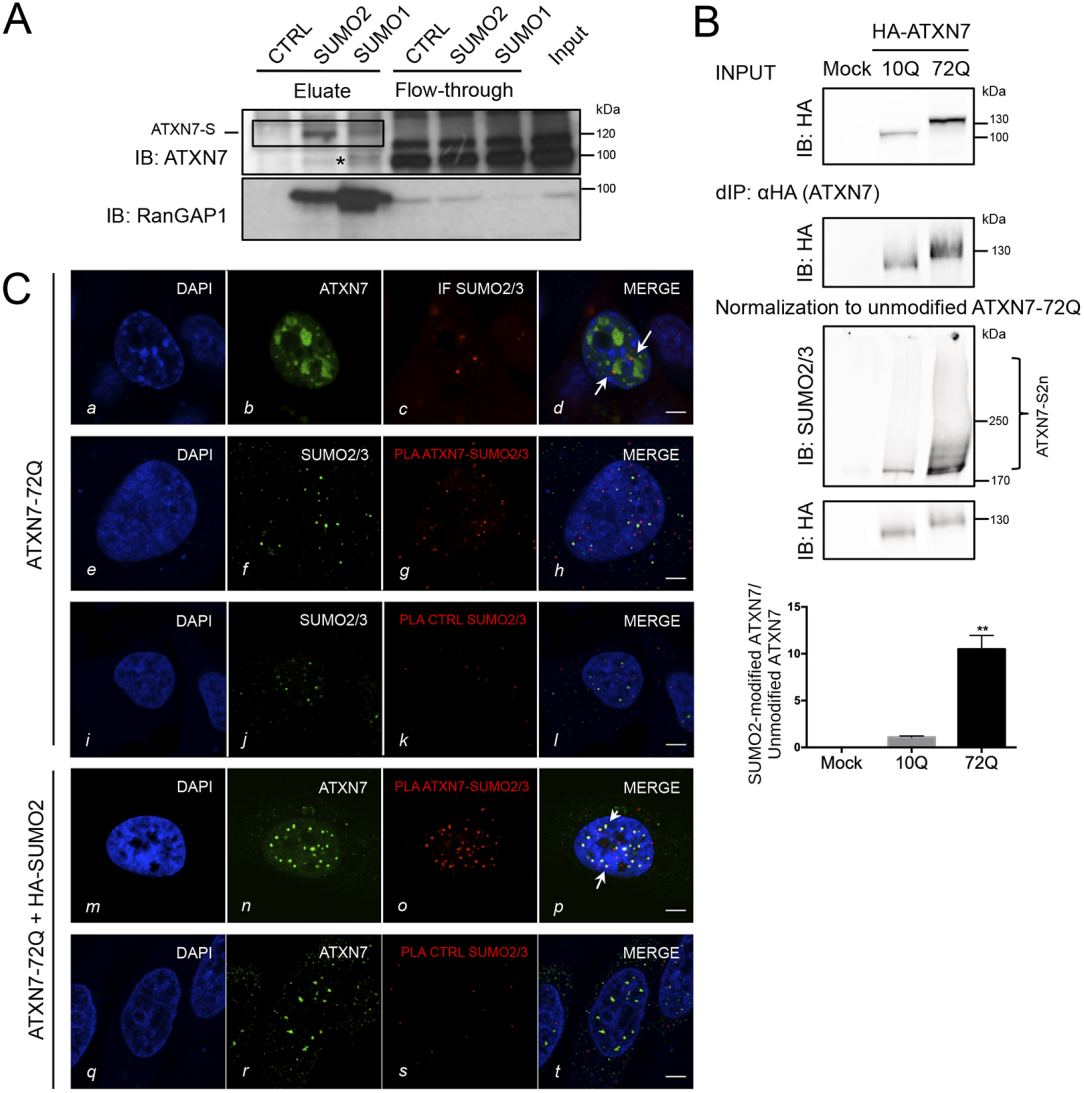
**ATXN7 is modified by SUMO2 in cells.** (A) MCF7 cell lysate was subjected to denaturing immunoprecipitations with beads coupled to monoclonal antibodies against SUMO1, SUMO2 or IgG (control). Top: enriched endogenous SUMO targets were eluted from beads with peptides corresponding to the epitopes of both SUMO antibodies. Shown are immunoblots against ATXN7 and against the abundant SUMO target RanGAP1 as positive control. SUMO-modified ATXN7 is boxed (ATXN7-S). The asterisk indicates non-specific band. (B) Both wild-type (10Q) and mutant (72Q) ATXN7 are SUMO2/3 modified. HEK293 cells expressing HA-ATXN7 with 10Q or 72Q were subjected to denaturing immunoprecipitation (d-IP) using anti-HA antibody-coupled beads (d-IP: HA), followed by western blotting. Input and d-IP products are revealed with anti-HA tag (top). To compare the level of SUMO2/3 modification, normalization to the unmodified protein is mandatory: d-IP products with a similar level of unmodified HA-ATXN7-10Q and 72Q were analyzed (IB: HA, bottom). Quantification of the SUMOylated species is shown (graph). Results are mean±s.d. Statistical analysis was performed using Student's *t*-test (**P<0.01; *n*=3). (C) Interaction between ATXN7-72Q and SUMO2/3 and their colocalization in HeLa cells determined by proximity ligation assay (PLA, antibodies used are written in red) and immunofluorescence. Row *a-d*: overexpressed ATXN7-72Q detected by immunofluorescence appears as irregular dots; some of them are colocalized or juxtaposed (arrows) with endogenous SUMO2/3. Row *e-h*: overexpressed ATXN7 and endogenous SUMO2/3: the red dots in the PLA assay demonstrate that mutant ATXN7 and endogenous SUMO2/3 are in close contact. By immunofluorescence (green dots), we detected ATXN7 and SUMO2/3. Row *m-p*: co-expression of mutant ATXN7-72Q and SUMO2 led to the complete colocalization between ATXN7 dots (green, immunofluorescence) and PLA foci (red). Arrows in *p* indicate examples of complete colocalization between ATXN7 immunofluorescence and the PLA signal. Mouse monoclonal anti-1C1 (ATXN7) and rabbit polyclonal anti-SUMO2/3 antibodies were used. Rows *i-l* and *q-t*: two negative controls for PLA show that no unspecific signal was detected (*k*, *s*). Representative confocal images are shown. Scale bars: 5 µm.

Modification of proteins with the different SUMO paralogs SUMO1 or SUMO2/3 produces different functional outcomes. Although we have previously shown that cellular ATXN7 is SUMOylated upon overexpression of SUMO1 (Janer et al., 2010), it remained unclear which SUMO paralog is conjugated at endogenous levels. Therefore, we performed immunoprecipitations using a protocol designed for the specific enrichment of endogenous SUMO1 and SUMO2/3-modified proteins from extracts prepared under denaturing conditions (Barysch et al., 2014) Using MCF7 cells, a cell line in which ATXN7 is well expressed, antibodies against both SUMO1 and SUMO2/3 could efficiently enrich endogenous modified ATXN7, producing bands at 120 kDa when probed with anti-ATXN7 antibody (Fig. 1A, top) and at 90 kDa when probed with anti-RanGAP1 antibody, used here as a control for anti-SUMO immunoprecipitation (Fig. 1A, bottom; Fig. S1). We conclude that endogenous ATXN7 can be conjugated by both SUMO1 and SUMO2/3.

For the polyQ-containing proteins huntingtin and ataxin-1, it has been shown that SUMO2 modification and subsequent proteasomal degradation modulates the accumulation of insoluble mutant protein (Guo et al., 2014; O'Rourke et al., 2013). As our interest is understanding the mechanism modulating accumulation of misfolded mutant ATXN7 and, further, given the involvement of SUMO2 in the degradation of polySUMOylated proteins, we decided to focus our investigations on the modification of polyQ-ATXN7 by SUMO2/3.

To determine whether endogenous SUMO2/3 is also conjugated to the pathogenic mutant ATXN7, we expressed wild-type (10Q) or mutant (72Q) hemagglutinin (HA)-tagged ATXN7 in HEK293 cells, followed by a denaturing immunoprecipitation using anti-HA-coupled beads. Mutant 72Q-HA-ATXN7 accumulates in cells compared with 10Q-HA-ATXN7, as attested by the intensity of the respective bands, both in input and after denaturing immunoprecipitation (Fig. 1B, top). Therefore, for a correct comparison of the SUMOylation levels between 10Q and 72Q-HA-ATXN7, we adjusted the amount of the 10Q and the 72Q ATXN7 proteins according to a similar level of unmodified ATXN7 (Fig. 1B, bottom, IB: HA**)** (Guo et al., 2014). We found both normal (10Q) and mutant (72Q) ATXN7 conjugated to endogenous SUMO2/3 (Fig. 1B, bottom, IB: SUMO2/3), migrating at high-molecular-mass (HMW) levels**.** These HMW bands were more intense for mutant (72Q) ATXN7 than for normal (10Q) ATXN7 and absent in mock-transfected cells. Densitometry scanning of blots and quantification showed that the SUMO2/3-modified fraction of mutant ATXN7 was almost 10-fold more abundant than its wild-type counterpart (Fig. 1B, graph).

To investigate the localization of mutant ATXN7 and SUMO2 in cell nuclei, we combined immunofluorescence and the proximity ligation assay (PLA), a technique that permits the detection of proteins located in close proximity (40 nm or less). Mutant ATXN7-72Q was expressed either alone or with HA-SUMO2 in HeLa cells. Overexpressed ATXN7-72Q can be detected by immunofluorescence in intranuclear inclusions (Fig. 1C, *b*) that appear as irregular dots of various sizes that are either juxtaposed or colocalized with SUMO2/3-positive dots that probably represent nuclear bodies. The presence of red dots in the PLA assay demonstrates that mutant ATXN7 and endogenous SUMO2/3 are in close contact in the nucleoplasm (Fig. 1C, *g*). Interestingly, the PLA signal did not always colocalize with endogenous SUMO2/3-positive dots visualized by immunofluorescence, suggesting that mutant ATXN7 interacts with endogenous SUMO2 also outside of nuclear bodies, in which SUMO2/3 is normally most concentrated.

However, co-expression of SUMO2 with ATXN7-72Q led to the complete colocalization between ATXN7 dots (green, immunofluorescence) and PLA foci (red), suggesting that a high degree of SUMOylation modified ATXN7 intranuclear localization (Fig. 1C, *n-p*). Overexpression of SUMO2 modifies the aspect of intranuclear accumulated ATXN7-72Q and leads to the formation of more numerous ATXN7-positive small dots. We conclude that mutant ATXN7 and endogenous or exogenously overexpressed SUMO2 are in close proximity in the nucleus, and that enhanced expression of SUMO2 impacts the subnuclear localization of ATXN7.

### ATXN7 inclusions colocalize with endogenous SUMO2/3, PML and ubiquitin

**Fig. 2.**
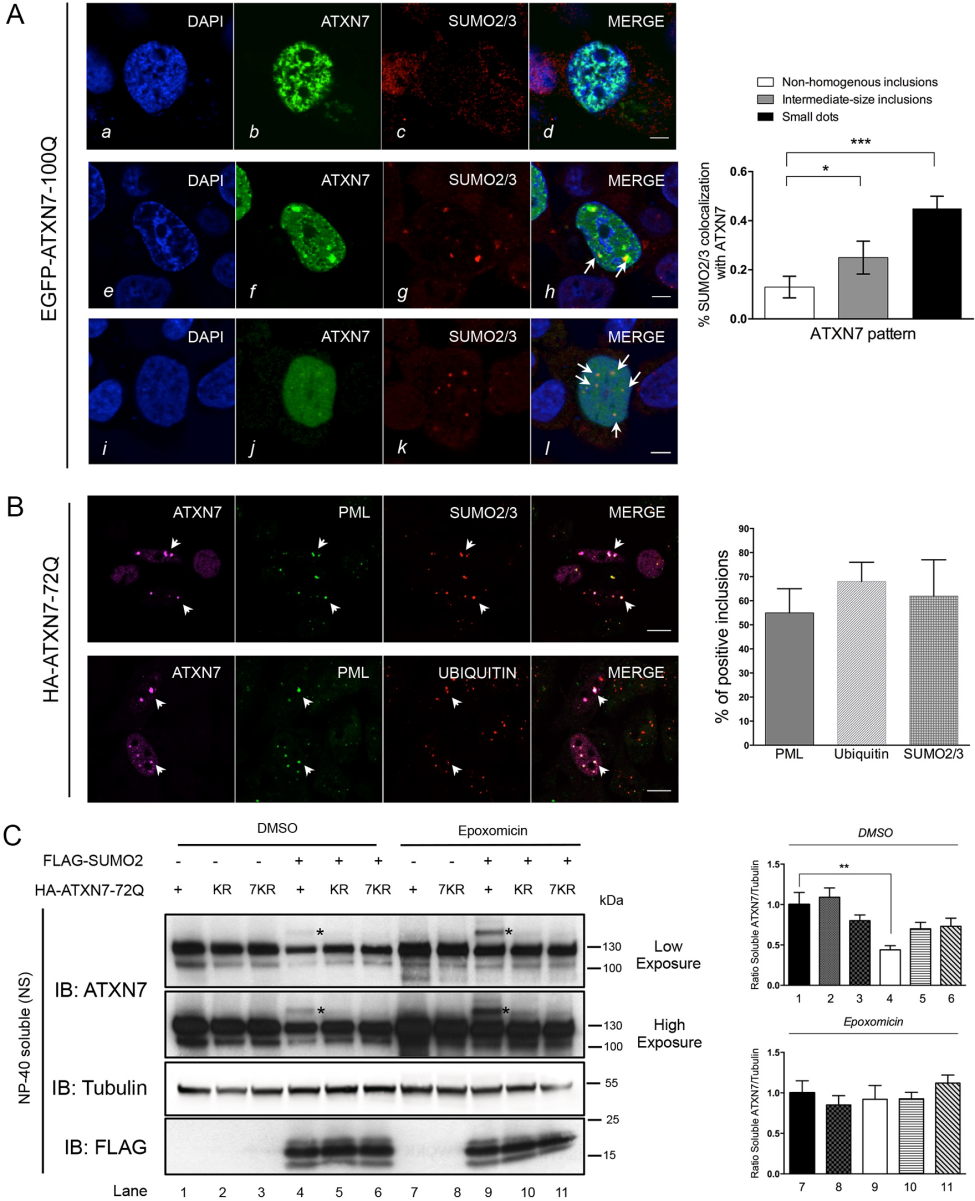
**Mutant ATXN7 aggregation and colocalization with SUMO2/3, PML and ubiquitin.** (A) In HeLa cells, overexpressed EGFP-ATXN7-100Q colocalized with endogenous SUMO2/3 in patterns reflecting the stage of ATXN7 aggregation: *a-d*, intensely stained, non-homogenous ATXN7-positive inclusions with rough edges did not colocalize with SUMO2/3; *e-h*, large ATXN7-positive intermediate-sized inclusions in which the mutant protein is juxtaposed to SUMO2/3 (*h*, arrows); *i-l*, diffuse nuclear ATXN7 and small mutant ATXN7-positive inclusions that colocalize with SUMO2/3-positive dots (*l*, arrows). A rabbit polyclonal anti-SUMO2/3 antibody was used. Representative confocal images are shown. Scale bars: 5 µm. The graph on the right shows the percentage of endogenous SUMO2/3 colocalized with each of the three ATXN7 patterns (see images, left) in *n*=65 cells from three independent experiments: three configurations were distinguished according to expression levels, i.e. the fractional area of ATXN7-positive inclusions relative to the nuclear surface of the confocal section. Results are mean±s.e.m. Statistical analysis was performed using Student's *t*-test: *P<0.015, ***P<0.0007. (B) In HeLa cells, overexpressed HA-ATXN7-72Q detected in inclusions colocalized with endogenous PML in nuclear bodies and with SUMO2/3. The ATXN7-72Q-positive inclusions also colocalized with ubiquitin and PML. Arrows highlight the colocalization of ATXN7 inclusions with the indicated proteins (PML and SUMO2/3, and PML and ubiquitin). Antibodies used: rabbit polyclonal anti-ATXN7, chicken anti-PML, mouse monoclonal anti-polyubiquitinated proteins FK2, mouse anti-SUMO2/3. Quantification of ATXN7-72Q-positive inclusions with PML, ubiquitin and SUMO2/3 in *n*=130 cells from *n*=3 independent experiments. Representative confocal images are shown in A (scale bars: 5 µm) and B (scale bars: 10 µm). (C) HEK293 cells overexpressing mutant ATXN7-72Q or its SUMO-deficient KR or 7KR mutants were analyzed in the presence of endogenous or overexpressed SUMO2. Shown are immunoblots of the NP-40-soluble fraction (NS) analyzed with anti-ATXN7 and anti-FLAG to detect expressed SUMO2. The proteasome was inhibited by epoxomicin (1 µM) treatment for 16 h. Asterisks indicate SUMO2-modified ATXN7-72Q. Quantification of the ratio of soluble ATXN7/tubulin, compared with the condition in lane 1 (DMSO, top graph) or lane 7 (epoxomicin, bottom graph). Data are mean±s.d. of *n*=3 independent experiments. Statistical analysis was performed using Student's *t*-test (**P<0.01).

We next investigated whether the interaction between SUMO2/3 and mutant ATXN7 correlated with aggregation. We overexpressed polyQ-expanded EGFP-ATXN7-100Q in HeLa cells and determined the localization of ATXN7 and endogenous SUMO2/3. Different populations of cells could be distinguished with respect to ATXN7 expression levels by the form or size of the proteinaceous inclusions in the nucleus and their colocalization with endogenous SUMO2/3. Cell nuclei with strong ATXN7 expression (23% of the total cells analyzed) contained large non-homogenous inclusions with little or no distinct SUMO2/3 staining (13% SUMO2/3-ATXN7 colocalization) (Fig. 2A, *b-d*). Cells with moderate ATXN7 expression levels (29% of total) presented intermediate-sized, or smaller, dense mutant ATXN7 inclusions, which contained or are juxtaposed to small dot-like SUMO2/3-positive structures (Fig. 2A, *f-h*; 25% SUMO2/3-ATXN7 colocalization). Finally, cells displaying lowest expression (48% of total) of ATXN7 contained mostly diffuse mutant ATXN7, with some concentration in small dots displaying increased colocalization (45%) with endogenous SUMO2/3 dots (Fig. 2A, *j-l*). These data suggest that colocalization between ATXN7 and endogenous SUMO2/3 correlates with the form and size of the proteinaceous inclusions. Interestingly, the smallest ATXN7 dots colocalize more often (45%) with endogenous SUMO2/3. Whether the correlation observed reflects how cells deal with misfolded mutant ATXN7 cannot be concluded here.

We next investigated whether mutant ATXN7-positive dots that colocalize with SUMO2/3 correspond to nuclear bodies and determined the ubiquitination status of ATXN7. We expressed HA-ATXN7-72Q in HeLa cells and triple-immunofluorescence staining showed that intranuclear inclusions of mutant ATXN7 colocalized extensively (55±10% of colocalization) with PML nuclear bodies (Fig. 2B) and widely with SUMO2/3 dots (62±15% of colocalization). We hypothesized that a SUMO-dependent ubiquitin ligase could act on mutant ATXN7, as was demonstrated for ATXN1 (Guo et al., 2014), and checked the ubiquitination status of mutant ATXN7 inclusions. Indeed, ATXN7-positive intranuclear dots colocalized to a high extent (68±8%) with ubiquitin (Fig. 2B). This demonstrates that mutant ATXN7 concentrates in dots that colocalize with PML bodies and SUMO2/3. In addition, mutant ATXN7 intranuclear inclusions colocalize with ubiquitin, suggestive of a possible link with degradation.

### SUMO2 modification of ATXN7 promotes protein degradation

Poly-SUMOylation by SUMO2 is known to be related to protein degradation (reviewed in Sriramachandran and Dohmen, 2014). Moreover, we have shown previously that clastosomes, which are PML bodies enriched in proteasomal subunits, degrade mutant ATXN7 (Janer et al., 2006). In order to investigate SUMO2 and a potential involvement of the proteasome, we compared the expression of mutant ATXN7-72Q and its SUMOylation-deficient variant K257R (KR) (Janer et al., 2010), as well as variant 7KR with seven lysines mutated into arginine at positions 117, 223, 226, 257, 287, 321 and 858, in HEK293 cells. We analyzed the NP-40-soluble (NS) fraction by western blotting.

Without SUMO2 co-expression, SUMOylated ATXN7-72Q was undetectable on direct immunoblots in the NS fraction (Fig. 2C, lane 1). Co-expression of SUMO2 resulted in weakly detectable levels of SUMOylated polyQ-ATXN7 (asterisks, lane 4). Finally, proteasome inhibition with epoxomicin led to a striking stabilization of the SUMOylated ATXN7-72Q (asterisks, lane 9). As expected, the SUMOylation-deficient variants KR and 7KR were not modified by SUMO2. In addition, the band corresponding to unmodified ATXN7 was significantly reduced (0.44±0.05) in the presence of SUMO2 co-expression (Fig. 2C, lane 4, compared with lane 1), an effect reversed by epoxomicin treatment. Taken together, these results suggest that SUMO2 modification contributes to ATXN7-72Q degradation, most probably via the proteasome.

### The SUMO-dependent ubiquitin ligase RNF4 mediates ATXN7 degradation via the proteasome

**Fig. 3.**
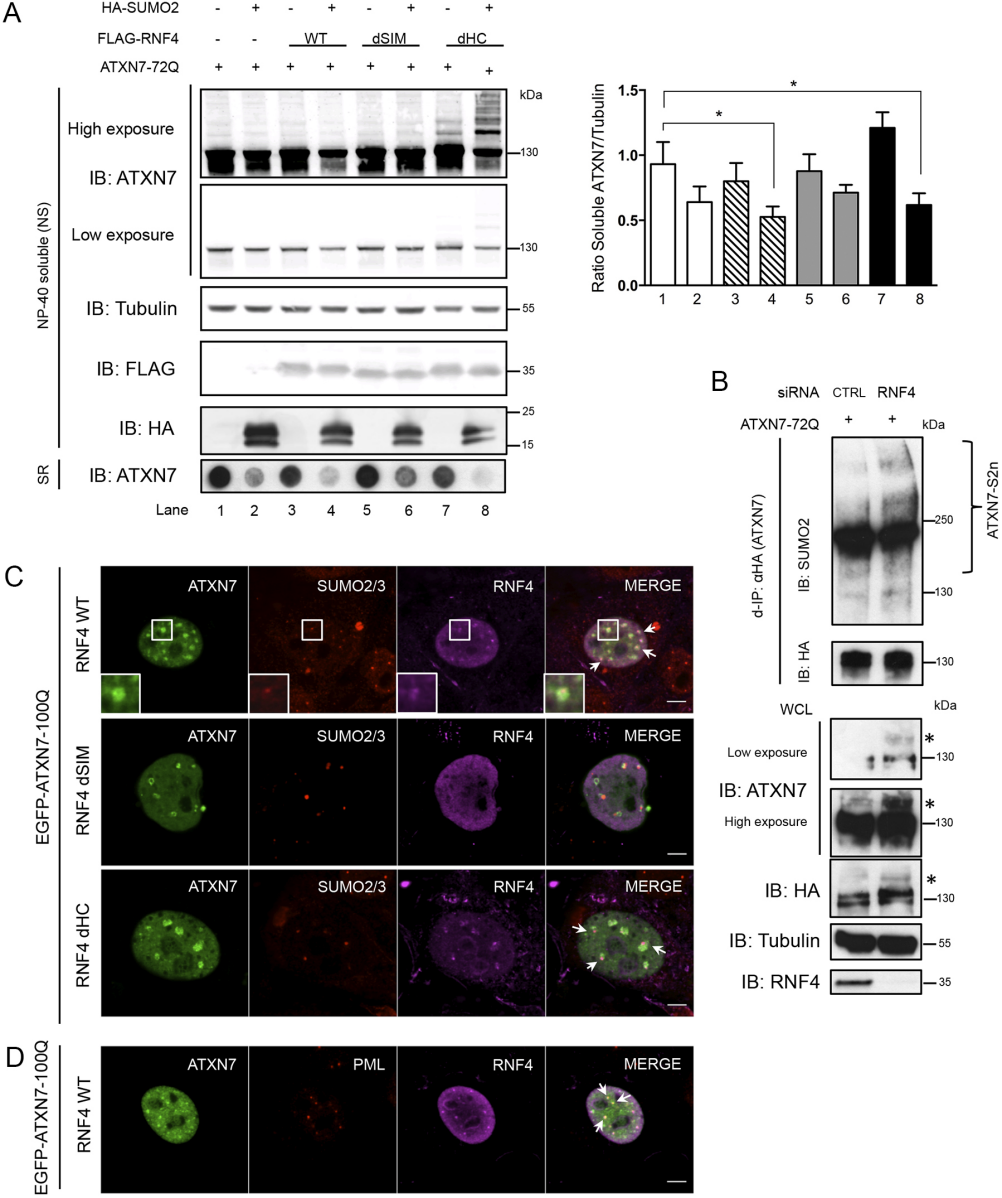
**SUMO-dependent ubiquitin ligase RNF4 mediates ATXN7 degradation.** (A) HeLa cells overexpressing ATXN7-72Q, SUMO2 and RNF4 [wild type (WT) or mutated in the SIM (dSIM) or RING domain (dHC)]: immunoblot analysis of the NP-40-soluble fraction (NS). Filter retardation assay of the SDS-resistant (SR) fraction (*n*=4). Quantification of the ratio of soluble ATXN7/tubulin expressed as mean±s.d. (*n*=3). Statistical analysis was performed using Student's *t*-test: *P<0.05 (lanes 4 and 8 compared with lane 1). (B) Levels of SUMOylated HA-ATXN7-72Q in cells treated with a control siRNA (CTRL) or an siRNA targeting RNF4 (*n*=2). Normalization: d-IP products with similar levels of unmodified ATXN7 protein were compared (d-IP, IB: HA). WCL, whole-cell lysate. ATXN7 was detected with two different antibodies (polyclonal anti-ATXN7 and monoclonal anti-HA) confirming that the HMW species (indicated by asterisks) are a modified form of ATXN7. (C) HeLa cells co-expressing EGFP-ATXN7-100Q and RNF4 [wild type (WT) or mutated in the SIM (dSIM) or RING domain (dHC)]: immunofluorescence shows RNF4 relative to SUMO2-positive ATXN7 intranuclear inclusions. RNF4 WT perfectly colocalized with mutant ATXN7 and SUMO2 (see arrows and higher magnification images); the RING mutant still colocalized (arrows) but the RNF4 SIM mutant did not and displayed only diffuse nuclear signal. (D) Colocalization between overexpressed mutant ATXN7, RNF4 and endogenous PML (arrows) in the nucleus of HeLa cells. Representative confocal images are shown. Scale bars: 5 μm.

Proteasomal degradation of poly-SUMOylated proteins has been shown to involve the action of the SUMO-dependent ubiquitin ligase RNF4 (Lallemand-Breitenbach et al., 2008; Rojas-Fernandez et al., 2014; Tatham et al., 2008; Ulrich, 2008). To test this idea, we expressed ATXN7-72Q in combination with wild-type RNF4 and RNF4 mutated in the SUMO-interacting motifs (SIMs) (to prevent binding to SUMO2 chains) or in the RING domain (to abolish ubiquitin E3 ligase activity) (Fig. 3A; Fig. S2). This was done with or without overexpression of SUMO2 in order not to limit SUMO2 levels. Co-expression of wild-type RNF4 with SUMO2 significantly decreased (0.52±0.08, lane 4) the level of soluble mutant ATXN7 compared with the expression of ATXN7-72Q only (0.93±0.17, lane 1). Expression of the RNF4 SIM mutant did not significantly influence the level of soluble ATXN7 (0.71±0.06, lane 6), but expression of the RNF4 RING mutant led to accumulation of SUMOylated ATXN7, an effect that was potentiated by co-transfection of SUMO2 (with decreased unmodified ATXN7, 0.61±0.09, lane 8). Thus, the RNF4 RING mutant appeared to behave as a dominant negative. This highly SUMOylated ATXN7 exhibits greater solubility, as decreased levels of sodium dodecyl sulfate (SDS)-resistant aggregates (SR) were detected in these cells by the filter retardation assay (Fig. 3A, bottom). This assay is complementary to western blotting as it detects aggregated species that do not enter the resolving gel because they are trapped in the stacking gel. Moreover, in the SR fraction, cells co-expressing SUMO2 and wild-type RNF4 contain fewer SDS-resistant aggregates than cells co-expressing only SUMO2. Together, our results on soluble and insoluble mutant ATXN7 suggest a role for RNF4 in misfolded mutant ATXN7 degradation. Consistent with these observations, silencing of endogenous RNF4 by short interfering RNA (siRNA) led to an increase in SUMOylated ATXN7 species, both after denaturing immunoprecipitation (Fig. 3B, top, IB: SUMO2) and in direct immunoblotting (see asterisks in Fig. 3B, bottom, IB: ATXN7 and IB: HA).

Analysis by three-color immunofluorescence (Fig. 3C) showed mutant ATXN7, RNF4 and endogenous SUMO2/3 to colocalize in numerous subnuclear foci. Although the RNF4 RING mutant (dHC) still colocalized with a subset of foci containing SUMO2/3 and ATXN7, the RNF4 SIM mutant did not, most probably due to its inability to recognize and bind SUMO2 chains (Fig. 3C). Given our previous demonstration that mutant ATXN7 is recruited into nuclear PML clastosomes, where it is degraded by proteasome components (Janer et al., 2006), we thus asked whether the modification by SUMO2 and the recognition by RNF4 contribute to the recruitment of mutant ATXN7 into PML bodies. Immunofluorescence staining of mutant ATXN7 showed indeed its colocalization with RNF4 protein within PML bodies (Fig. 3D). Together, our results suggest that the SUMO2-mediated degradation of mutant ATXN7 involves the action of RNF4 and thus provides a molecular explanation (Gärtner and Muller, 2014) for the previously described pathway for ATXN7 degradation in PML clastosomes (Janer et al., 2006).

**Fig. 4.**
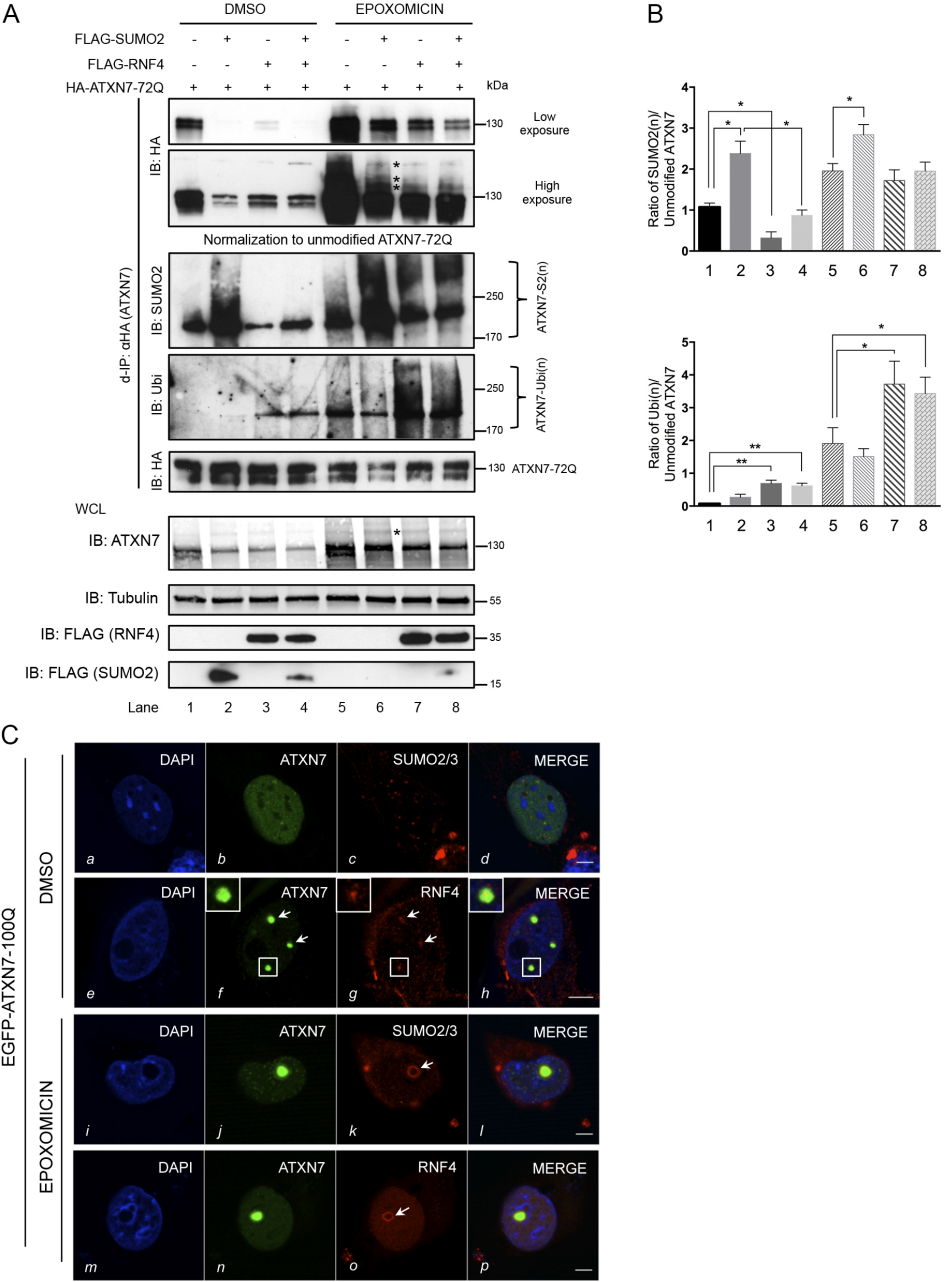
**Proteasome inhibition abolishes mutant ATXN7 degradation via RNF4.** (A) HEK293 cells overexpressing ATXN7-72Q, SUMO2 and RNF4 were treated with DMSO (control) or epoxomicin (1 µM for 16 h). Denaturing immunoprecipitation (d-IP) samples with similar levels of unmodified ATXN7-72Q were analyzed, as normalization is required for a comparison of modified ATXN7 levels. d-IP products, before normalization (top panel, low and high exposures; asterisks indicate poly-SUMO2 modified HA-ATXN7-72Q), and input in whole-cell lysate (WCL; bottom panel; asterisk indicates SUMO2-modified ATXN7) are shown. d-IP products after normalization are shown in the middle panel (IB: SUMO2, IB: Ubi, IB: HA). Co-expression of RNF4 and SUMO2 decreased SUMO2-modified ATXN7. Epoxomicin treatment led to SUMO2- (IB: SUMO2) and ubiquitin- (IB: Ubi) modified ATXN7 accumulation. RNF4 promoted the ubiquitination of modified ATXN7. (B) Top: quantification of the ratio of polySUMOylated ATXN7-S2(n)/unmodified HA-ATXN7-72Q in DMSO (lanes 1-4) and epoxomicin conditions (lanes 5-8). Bottom: quantification of the ratio of polyubiquitinated ATXN7-Ubi(n)/unmodified HA-ATXN7-72Q. Data are mean±s.d. (*n*=3). Statistical analysis was performed using Student's *t*-test: *P<0.05, **P<0.01. (C) Colocalization of expressed EGFP-mutant ATXN7 with endogenous SUMO2/3 and endogenous RNF4 in HeLa cells in control (DMSO, *a-h*) or epoxomicin (*i-p*) conditions. Endogenous RNF4 was detected in a subset of ATXN7-positive inclusions in the control condition (*f*, *g*, see arrows and higher magnification images), but following epoxomicin treatment became enriched around one large EGFP-ATXN7-100Q inclusion (*o*, arrow). SUMO2/3 shows a similar enrichment around an ATXN7 inclusion when proteasomal degradation is blocked (*k*; the arrow points to endogenous SUMO2/3 surrounding one large EGFP-ATXN7-100Q inclusion in epoxomicin-treated cells). Representative confocal images are shown. Scale bars: 5 μm.

To investigate whether the RNF4-induced decrease in mutant ATXN7 was due to proteasome-dependent degradation, we inhibited the proteasome by epoxomicin in HEK293 cells co-expressing ATXN7-72Q and wild-type RNF4 with or without overexpression of SUMO2, followed by denaturing ATXN7 immunoprecipitation. In order to determine the extent to which ATXN7 is SUMO or ubiquitin modified, samples need to be normalized with respect to the levels of unmodified ATXN7 (see explanation provided with Fig. 1B). Results are presented before and after normalization. Consistent with our previous results (Fig. 2C), proteasome inhibition by epoxomicin led to a strong accumulation of SUMO2-conjugated ATXN7 [Fig. 4A, d-IP: HA (ATXN7); IB: SUMO2, lanes 5-8] by a factor of 1.95 (Fig. 4B, top, lane 5). In dimethyl sulfoxide (DMSO) conditions, overexpression of RNF4 leads to a significant decrease in SUMO2(n)-modified ATXN7 (Fig. 4B, top; 0.32±0.15, lane 3 compared with lane 1), in agreement with our hypothesis on the role of RNF4 in ATXN7 degradation. Additionally, following denaturing immunoprecipiation, samples were analyzed for their polyubiquitin content (IB: Ubi). In control (DMSO) samples, RFN4 expression enhances almost six times, from 0.1 to 0.62, the polyubiquitin signal in normal condition (Fig. 4B, bottom, compare lanes 3-4 with lane 1). After epoxomicin treatment, there is a strong (1.910±0.480, lane 5) accumulation of Ubi-conjugated ATXN7, an effect that is highly triggered by RNF4 (3.72±0.70, lane 7; 3.43±0.50, lane 8) (Fig. 4B, bottom), suggesting that RNF4 expression strongly enhances the polyubiquitination of SUMO2-modified ATXN7. Furthermore, in DMSO samples, RNF4 co-expressed with SUMO2 leads to a strong decrease in SUMO-modified ATXN7, compared with SUMO2 expression only (Fig. 4B, top, lane 4 compared with lane 2), an effect that is reversed in the presence of epoxomicin. Altogether, these results strongly suggest the involvement of RNF4 in the clearance of poly-SUMO2 modified mutant ATXN7 via the proteasome.

### Endogenous RNF4 colocalizes with ATXN7 and SUMO2

We then compared, by immunofluorescence, the cellular distribution of endogenous and exogenous RNF4 in cells expressing EGFP-ATXN7-100Q. Exogenous wild-type RNF4 was both diffuse and colocalized with nuclear EGFP-ATXN7-100Q inclusions (Fig. 3C), whereas endogenous RNF4 was detected only within the larger-sized EGFP-ATXN7-100Q inclusions in ATXN7-overexpressing cells (Fig. 4C, *g*). Following proteasome inhibition, endogenous RNF4 became enriched around a single large EGFP-ATXN7-100Q inclusion in a subset of cells (Fig. 4C, *o*). A similar pattern was observed for SUMO2/3 in epoxomicin-treated cells (Fig. 4C, *k*). These findings are consistent with a possible role of RNF4 in mediating the SUMO-dependent degradation of ATXN7.

### Investigation of the SUMO pathway in SCA7 knock-in mice

To determine whether our previous findings could be transposed to an *in vivo* pathological context, we next investigated ATXN7 and SUMO2/3 colocalization in *Atxn7^100Q/5Q^* mice, a polyQ-ATXN7 knock-in mouse line that develops retinal degeneration, weight loss, kyphosis, ataxia, ptosis, tremor and gradual loss of mobility (Chen et al., 2012). Owing to instability of the CAG repeat, which leads to an expansion of 120Q on one allele, the lifespan of *Atxn7^100Q/5Q^* mice in our colony is 13-14 months, instead of 18 months (Chen et al., 2012). We analyzed cerebellum and retina, the most affected tissues in SCA7, at 12 months, when the mice are severely affected.

**Fig. 5.**
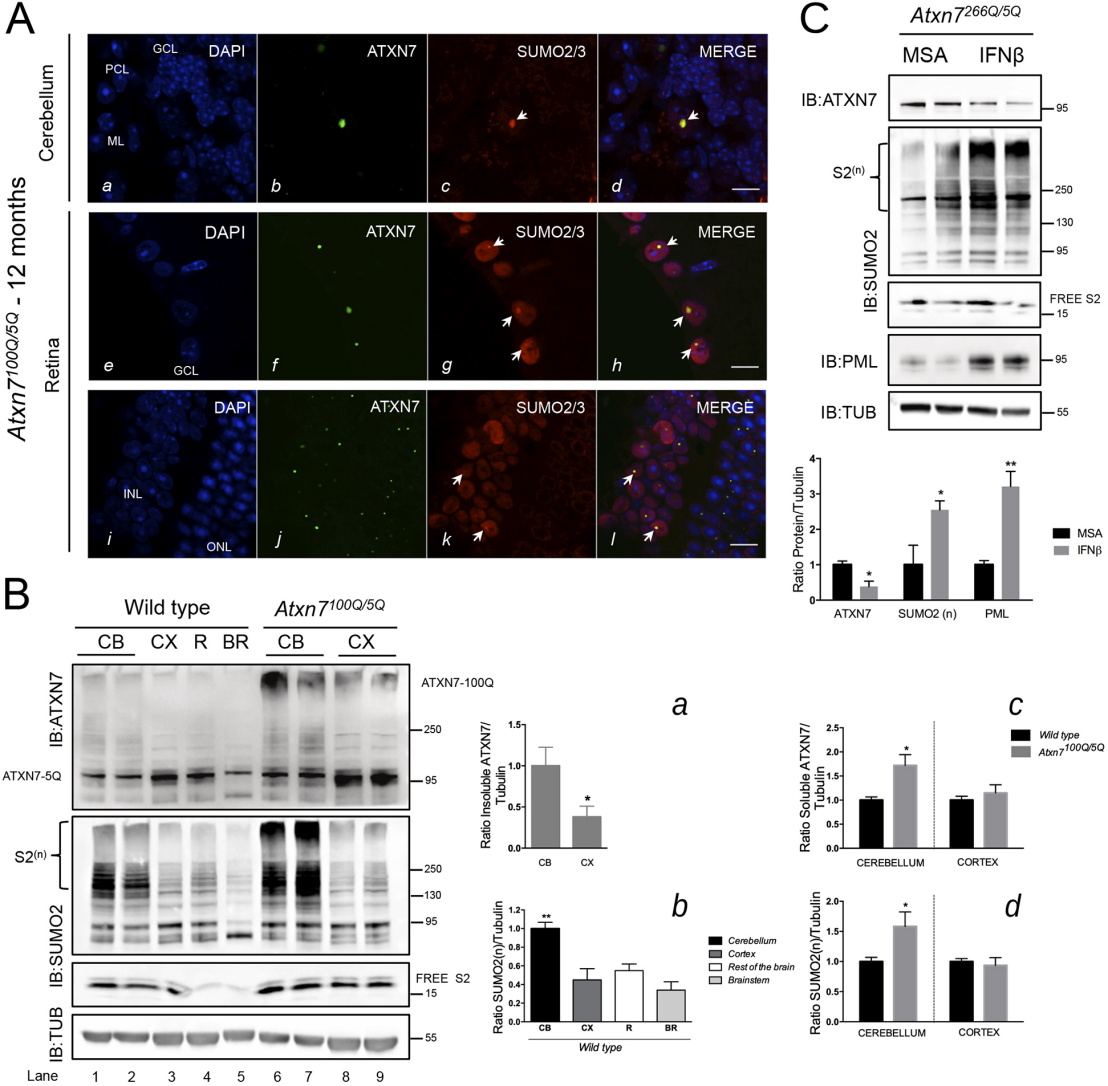
***In vivo* investigation of mutant ATXN7, SUMO2/3 and PML protein expression and colocalization in two SCA7 KI mouse models.** (A) Cerebellum (*a-d*) of a 12-month-old *Atxn7^100Q/5Q^* mouse was labeled with a polyclonal anti-ATXN7 antibody and a monoclonal anti-SUMO2 antibody. SUMO2 (dot in *c*, see arrow) colocalized strongly (arrow in *d*) with an ATXN7-positive intranuclear inclusion in a Purkinje cell (92.8±4.7% of colocalization; *n*=200 Purkinje cells counted in lobes V and VI from *n*=2 *Atxn7^100Q/5Q^* mice). GCL, granular cell layer; ML, molecular layer; PCL, Purkinje cell layer. Retina (*e-l*) of a 12-month-old *Atxn7^100Q/5Q^* mouse: SUMO2 protein (*g*, *k*, arrows indicate dots where SUMO2 is concentrated) colocalized perfectly with mutant ATXN7 inclusions in the ganglion cell layer (*h*, arrows) and in a subset of cells in the INL (*l*, arrows). GCL, ganglion cell layer; INL, inner nuclear layer; ONL, outer nuclear layer. Representative confocal images are shown. Scale bars: 10 μm. (B) Western blot analysis of brains from 12-month-old *Atxn7^100Q/5Q^* mice and their wild-type littermates. Levels of ATXN7 and poly-SUMO2 proteins are highly increased in the cerebellum of *Atxn7^100Q/5Q^* mice compared with wild-type littermates. Quantification of soluble ATXN7/tubulin and SUMO2(n)/tubulin is shown (graphs *c* and *d*; comparison of *Atxn7^100Q/5Q^* and wild-type mice)*.* In *Atxn7^100Q/5Q^* mice, mutant insoluble ATXN7 accumulates more in the cerebellum (1.00±0.23) than in cortex (0.38±0.13) (graph *a*). In wild-type littermates, poly-SUMO2 protein is more abundant in the cerebellum than in the cortex, rest of the brain and brainstem (graph *b*). Data are mean±s.d. from *n*=3 independent analyses. Statistical analysis was performed using Student's *t*-test: *P<0.05; **P<0.01. (C) Western blot analysis of cerebellar extracts from IFN-beta-treated *Atxn7^266Q/5Q^* mice compared with MSA-treated mice. An important increase (factor of 2.55±0.25) in the ratio of polySUMO2/tubulin was detected when comparing IFN-beta-treated with MSA-treated *Atxn7^266Q/5Q^* mice. In agreement with our previously published results (Chort et al., 2013), an increase in PML levels (factor of 3.2±0.42) and a decrease in soluble ATXN7 (by 60%) are observed in *Atxn7^266Q/5Q^* mice after IFN-beta treatment compared with MSA treatment. Data are mean±s.d. from *n*=3 independent analyses. Statistical analysis was performed using Student's *t*-test: *P<0.05; **P<0.01.

In the cerebellum of *Atxn7^100Q/5Q^* mice, ATXN7 is localized in the nucleus of Purkinje cells, where it mostly accumulates in a single large inclusion. SUMO2/3 colocalized with mutant ATXN7 in the inclusion (Fig. 5A). Retinal neurons are distributed in three nuclei layers: (1) the cell bodies of rod and cone photoreceptors that are in the outer nuclear layer (ONL); (2) the nuclei of bipolar, horizontal and amacrine cells that are in the inner nuclear layer (INL); and (3) nuclei of the ganglion cell layer (GCL), the closest to the optic nerve. Mutant ATXN7 accumulated in nuclear inclusions in *Atxn7^100Q/5Q^* mice retina. The inclusions' sizes differed depending on the retinal layer in which they were located: the larger inclusions were found in the GCL and colocalized perfectly with SUMO2 (Fig. 5A, *f-h*). By contrast, in the INL and ONL, where ATXN7-positive inclusions were smaller, SUMO2 staining was intense in INL nuclei but colocalization was only partial (Fig. 5A, *j-l*). Finally, in the ONL, SUMO2 staining was weak, mostly cytoplasmic and did not colocalize with ATXN7. Taken together, these results show significant co-occurrence of ATXN7 and SUMO2/3 in some (GCL), but not all (ONL), cells affected by SCA7 pathology in the *Atxn7^100Q/5Q^* mouse model. There are cell-type-specific differences that might explain the differences in the degree of colocalization between inclusions and SUMO2.

We then compared severely affected *Atxn7^100Q/5Q^* mice and wild-type littermates at 12 months of age, for their SUMO2 and ATXN7 content in affected brain regions, such as the cerebellum and brainstem, as well as in the less-affected cortex. Normal ATXN7 (5Q) is detected as a band around 97 kDa, whereas mutant ATXN7 in *Atxn7^100Q/5Q^* mice is SDS insoluble and detected in the stacking gel; its accumulation is more pronounced in the cerebellum (Fig. 5B, *a*; insoluble ATXN7/tubulin ratio of 1.0±0.23) than in cortex (ratio of 0.38±0.13). Additionally the level of soluble ATXN7 in the cerebellum was much higher (1.72±0.22) in *Atxn7^100Q/5Q^* mice than in their wild-type littermates (Fig. 5B, *c*).

Free SUMO2 is detected at 17 kDa (Fig. 5B, IB: SUMO2, free S2), whereas polySUMOylated proteins migrate as HMW species, up to more than 250 kDa [see S2(n)]. In wild-type mice, we quantified polySUMO2/3 species, which were highly enriched in the cerebellum (ratio of SUMO2/tubulin set at 1.0) compared with cortex (ratio of 0.45±0.12) and brainstem (ratio of 0.34±0.09) (Fig. 5B, *b*). A similar pattern was observed in *Atxn7^100Q/5Q^* mice. We next compared the polySUMO2/3 levels in the cerebellum between wild-type and *Atxn7^100Q/5Q^* mice and determined that SCA7 mice contain 1.58±0.23 times more SUMO2(n)/tubulin than the wild-type littermates. This suggested that, in severely affected mice, there is an accumulation of polySUMO2/3 species in the cerebellum compared with control littermates. This difference was not detected in the cortex, when comparing wild-type with *Atxn7^100Q/5Q^* mice, suggestive of a tissue- and disease-specific SUMO2/3 response. Whether the ATXN7 aggregates detected on western blots sequester SUMO2/3 and correspond to colocalized ATXN7 and SUMO2-positive nuclear inclusions (Fig. 5A) cannot be concluded here.

We previously showed that interferon (IFN)-beta stimulated the formation of PML clastosomes *in vivo*, a potential therapeutic approach for SCA7 (Chort et al., 2013). IFN-beta treatment of *Sca7^266Q/5Q^* mice reduced neuronal intranuclear inclusions composed of insoluble mutant ATXN7, in parallel to increased PML protein expression in nuclear bodies (Chort et al., 2013). By western blot analysis of IFN-beta-treated *Sca7^266Q/5Q^* mice (annotated in Fig. 5C as *Atxn7^266Q/5Q^*), we detected also an increase in PML levels (factor of 3.2±0.42) and a decrease in soluble ATXN7 (by 60%) (Fig. 5C) compared with levels in murine serum albumin (MSA)-treated mice. Importantly, IFN-beta improved the locomotion of *Sca7^266Q/5Q^* mice, demonstrating a therapeutic effect. It was demonstrated that IFN-alpha and -gamma dramatically increase general SUMO1 and SUMO2/3 levels, *in vitro* as well as *in vivo*, through a microRNA-based mechanism (Sahin et al., 2014): this result prompted us to analyze the SUMO2 levels in *Sca7^266Q/5Q^* knock-in mice treated with IFN-beta compared with MSA-treated littermates. Interestingly, we detected an important increase (factor of 2.55±0.25) in the ratio of polySUMO2/tubulin when comparing IFN-beta-treated with MSA-treated *Sca7^266Q/5Q^* mice (Fig. 5C). Thus, we suggest that, although IFN acts through many mechanisms, the degradation of mutant ATXN7 that we observed might involve the polySUMO2-modification of ATXN7.

**Fig. 6.**
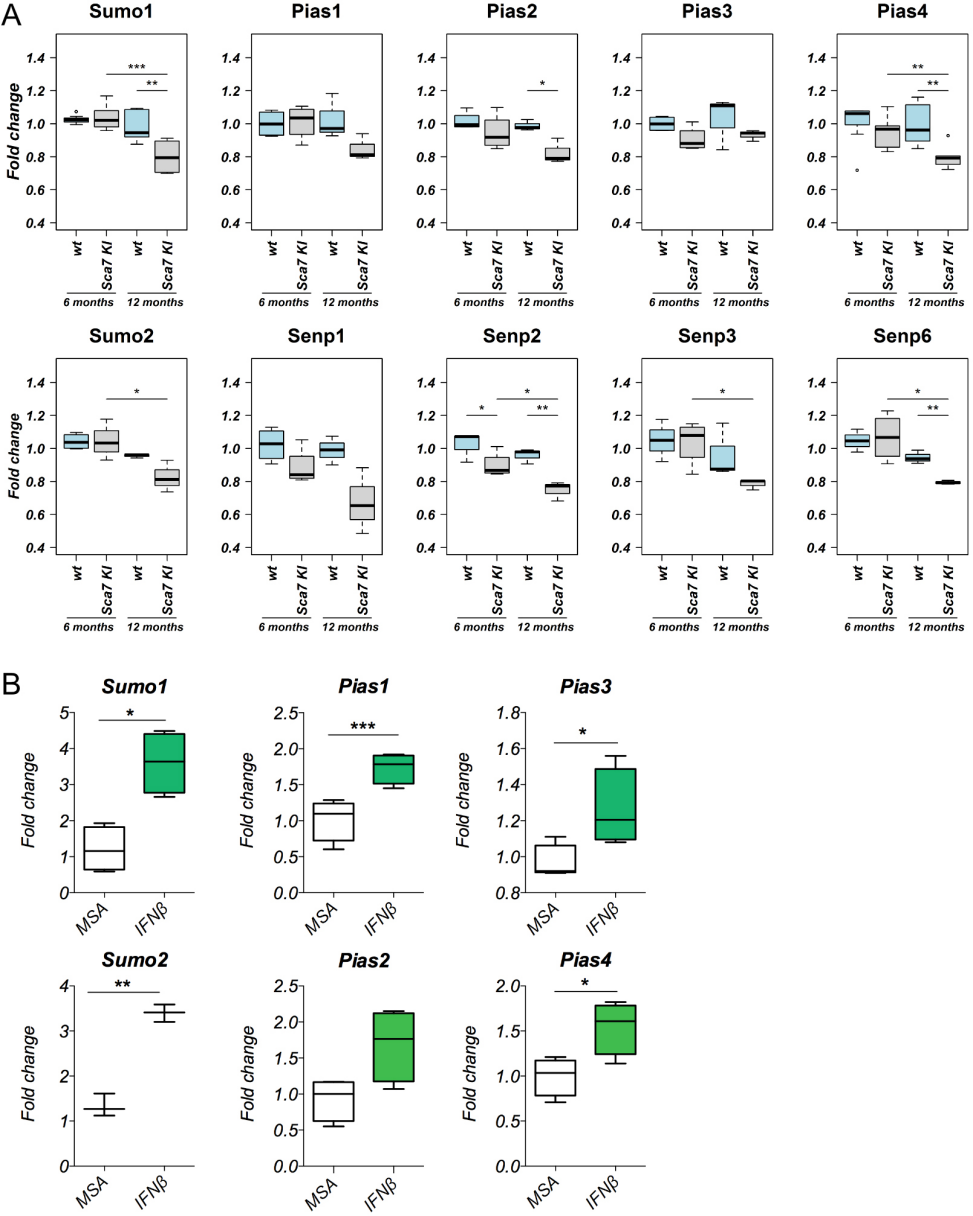
**Expression of SUMO pathway-related genes in two SCA7 knock-in mice models.** (A) Quantitative RT-PCR analysis of SUMO pathway enzymes in cerebella from 6- and 12-month-old *Atxn7^100Q/5Q^* mice and wild-type littermates. SUMO enzymes were differentially expressed in 6-month-old *Atxn7^100Q/5Q^* versus wild-type mice; a statistically significant decrease was observed only for *Senp2* (*P*=0.047). At 12 months, a statistically significant decrease in *Sumo1* (*P*=0.0048), *Pias2* (*P*=0.028), *Pias4* (*P*=0.024), *Senp2* (*P*=0.0079) and *Senp6* (*P*=0.0021) was observed. Comparison between 6-month-old and 12-month-old *Atxn7^100Q/5Q^* mice confirmed the significant decrease in *Sumo1* (*P*=0.0002), *Sumo2* (*P*=0.035), *Pias4* (*P*=0.007), *Senp2* (*P*=0.038), *Senp3* (*P*=0.027) and *Senp6* (*P*=0.023) mRNA expression. Samples were analyzed in triplicate and normalized to mouse *Rplp0* and *B2m*. (B) Quantitative RT-PCR of SUMO mRNAs from cerebella of 12 week-old *SCA7^266Q/5Q^* knock-in mice, injected intraperitoneally with either mouse IFN-beta or MSA (control). SUMO enzymes were significantly increased in IFN-beta-treated mice versus MSA controls: *Sumo1* (*P*=0.0158), *Sumo2* (*P*=0.0097), *Pias1* (*P*=0.0009), *Pias3* (*P*=0.0183), *Pias4* (*P*=0.0133). Samples were analyzed in triplicate and normalized to mouse *Rplp0* ribosomal gene. One-way ANOVA was applied for statistical analysis. Data are mean±s.d. (*n*=5 in each group). *P<0.05; **P<0.005; ***P<0.0005.

We next analyzed the expression of SUMO-pathway-related transcripts in the cerebellum of wild-type *Atxn7^5Q/5Q^* and *Atxn7^100Q/5Q^* mice at 6 and 12 months of age by quantitative reverse transcription polymerase chain reaction (RT-PCR). The relative levels of mRNA were standardized to *Rplp0* ribosomal RNA, and to beta-2microglobulin (*B2m*) as non-variant RNA species, according to Boda et al. (2009). At 6 months (before the development of motor symptoms), we did not observe statistically significant modifications in mRNA expression in the cerebella of *Atxn7^100Q/5Q^* mice compared with wild-type littermates, except for *Senp2* (Fig. 6A). At 12 months, a statistically significant reduction in *Sumo1* (*P*=0.0048), *Pias2* (*P*=0.028), *Pias4* (*P*=0.024), *Senp2* (*P*=0.0079) and *Senp6* (*P*=0.0021) expression was detected in *Atxn7^100Q/5Q^* mice compared with wild-type littermates (Fig. 6A). *Sumo2* expression was decreased at 12 months in *Atxn7^100Q/5Q^* mice compared with wild-type littermates, but this difference was not statistically significant. We also compared the differences in expression at 6 and 12 months of age in the *Atxn7^100Q/5Q^* mice and found a statistically significant difference in the expression of *Sumo1* (*P*=0.0002), *Pias4* (*P*=0.007), *Sumo2* (*P*=0.035), *Senp2* (*P*=0.038), *Senp3* (*P*=0.027) and *Senp6* (*P*=0.023), indicating that when disease worsens in mice, the SUMO pathway is downregulated.

We also analyzed mRNA expression of SUMOylation enzymes in *SCA7^266Q/5Q^* knock-in mice treated with IFN-beta compared with MSA-treated littermates. The results obtained were in line with our data on SUMO2/3 from western blot analyses: IFN-beta significantly increased the expression of *Sumo1* (*P*=0.0158), *Sumo2* (*P*=0.0097) and SUMO-related enzymes such as *Pias1* (*P*=0.0009), *Pias3* (*P*=0.0183) and *Pias4* (*P*=0.0133) in these mice (Fig. 6B), thus supporting the therapeutic benefit of IFN-beta treatment and confirming the importance of SUMOylation pathway in SCA7 pathology.

### Accumulation of SUMO pathway components in the cerebellum of SCA7 patients

**Fig. 7.**
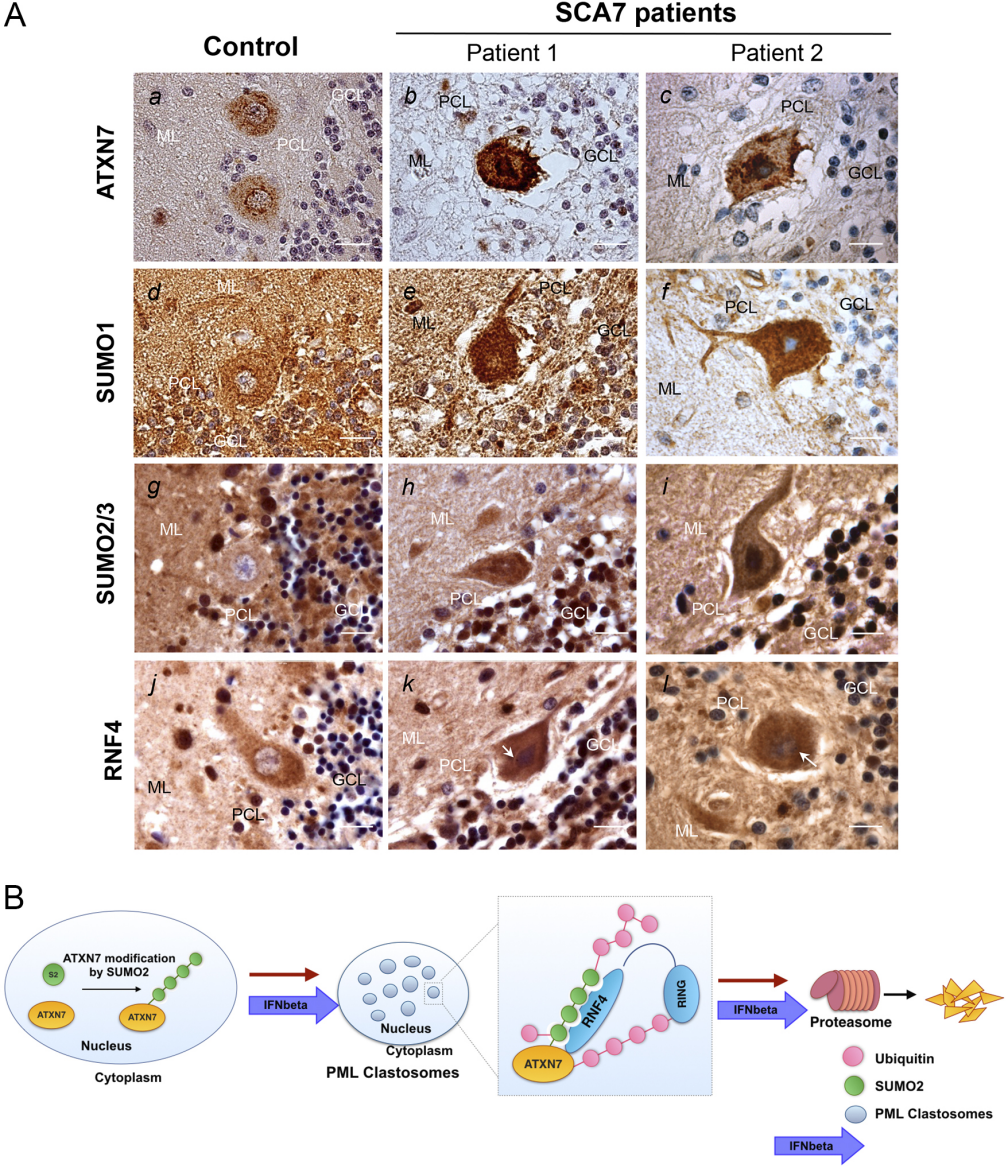
**SUMO proteins accumulate abnormally in the cerebellum of two SCA7 patients.** Model proposed for mutant ATXN7 degradation via the proteasome. (A) Postmortem cerebellar tissues from two SCA7 patients with morphologically and genetically confirmed SCA7 (57 and 10 years old at death; with 47 CAG repeats and 85 CAG repeats on the mutant allele in peripheral blood, respectively) and a control (52 year-old) with no neurological disease were analyzed by immunohistochemistry; representative images are shown. In the cerebellum of the SCA7 patients, ATXN7 accumulated strongly in nuclei and cytoplasm of the few surviving Purkinje cells (*b*, *c*). SUMO1 (*e*, *f*) and SUMO2/3 (*h*, *i*) immunoreactivity increased strongly in the nucleus and, to a lesser extent, in the cytoplasm of Purkinje cells in the SCA7 patients. RNF4 accumulated in the SCA7 patients, both in the cytoplasm and in the nucleus of the Purkinje cells (*k*, *l*). We analyzed *n*=20 Purkinje cells for each patient: cells in which nuclei were not visible were not taken into account. GCL, granular cell layer; ML, molecular layer; PCL, Purkinje cell layer. Scale bars: 20 μm. (B) Model for mutant ATXN7 degradation via the proteasome. We propose this model, which summarizes data from this study and our previously published results on polyQ-ATXN7 degradation by PML clastosomes and their stimulation by IFN-beta treatment (Janer et al., 2006; Chort et al., 2013). First, mutant ATXN7 is modified by SUMO2 by addition of poly-SUMO2/3 chains. This modification promotes the recruitment of ATXN7 to PML clastosomes, where it is ubiquitinated by the SUMO-dependent ubiquitin ligase RNF4 for its final degradation by the proteasome. Treatment with IFN-beta enhances clastosome formation and increases SUMO pathway enzymes, promoting further poly-SUMOylation by SUMO2/3 of polyQ-expanded ATXN7, which finally leads to its enhanced clearance.

Because neuronal death is particularly important in the Purkinje cell layer of SCA7 patients, we investigated SUMOylation of ATXN7 in the cerebellum of postmortem brains from two patients and a control. In the control brain, ATXN7 staining was diffuse in nuclei and the cytoplasm, with a stronger signal along the nuclear membrane (Fig. 7A, *a*). In the SCA7 patients, Purkinje cell bodies were shrunken and ATXN7 strongly accumulated in both the nuclei and cytoplasm (Fig. 7A, *b*, *c*). SUMO1 staining, which was granular and diffuse, increased in Purkinje cell nuclei and cytoplasm in the SCA7 brains, compared with the control brain, in which staining, mostly cytoplasmic, was also diffuse (Fig. 7A, *d* compared with *e*, *f*). SUMO2 was faintly stained in nuclei of Purkinje cells in the control brain, whereas in the two SCA7 cases, the shrunken Purkinje cell soma showed accumulation of SUMO2 in darker spots and the nuclei appeared condensed and strongly stained (Fig. 7A, *g* compared with *h*, *i*). RNF4 staining in the control brain was granular and diffuse, and mostly cytoplasmic in Purkinje cells (Fig. 7A, *j*). In the SCA7 brains, RNF4 staining was more intense than in the control brain; in addition, the nuclei of Purkinje cells were strongly labeled (Fig. 7A *k*, *l*). The accumulation of RNF4 in SCA7 Purkinje cells paralleled SUMO2 accumulation in the nucleus, suggesting deregulation of this pathway during the disease.

## DISCUSSION

Protein misfolding is a major dysfunction in polyglutamine diseases. The way neurons handle protein aggregates, either by refolding the proteins with molecular chaperones or eliminating them once formed, determines cell survival (Ross and Poirier, 2005).

We previously showed that SUMOylation reduced the toxicity and aggregation of exogenous mutant ATXN7 (Janer et al., 2010), but the question remained whether endogenous ATXN7 in proliferating cells is SUMO modified in physiological conditions. Here, we show, for the first time, that in unstressed cells, endogenous ATXN7 can be SUMOylated by SUMO1 and especially SUMO2/3. Interestingly, this is in agreement with SUMOylation of the yeast homolog of ATXN7, Sgf73, that was detected in a global proteomics analysis of SUMOylation (Wohlschlegel et al., 2004).

We also previously demonstrated the specificity of PML isoform IV and the role of clastosomes, specialized nuclear bodies enriched in proteasomes, in the degradation of mutant ATXN7 (Janer et al., 2006). In this study, we investigated the SUMO pathway and showed a colocalization of mutant ATXN7 and RNF4 within PML-positive nuclear bodies. Moreover, endogenous RNF4 was found accumulated around ATXN7 nuclear inclusions. We previously co-immunoprecipitated PML IV and mutant ATXN7 (Janer et al., 2006), which is also in agreement with the colocalization between mutant ATXN7 inclusions and PML observed in this study. Taken together, these data suggest that RFN4 is involved in the degradation of mutant ATXN7 within clastosomes. Indeed, our results are consistent with an interaction taking place within PML bodies and particularly in clastosomes, where proteasomal degradation follows the polyubiquitination of mutant ATXN7 by RNF4: a potential model is presented in Fig. 7B. RNF4-mediated protein degradation was first described for SUMO-modified PML degradation induced by arsenic (Lallemand-Breitenbach et al., 2008; Tatham et al., 2008). The colocalization between endogenous and expressed RNF4 (SNURF) and PML, along with its interaction in a complex being facilitated by SUMO1, was first shown by Häkli et al. (2005). Another transcription factor, Nrf2, which is degraded in the cytoplasm via Cul3/RING box1 E3 ubiquitin ligase, is also transferred to PML bodies when SUMOylated (Malloy et al., 2013). Overexpression of RNF4 decreased the levels of Nrf2 in a PML-enriched cell fraction, another example of how transcription factors can be degraded via the proteasome in both the cytoplasm and the nucleus (Malloy et al., 2013). We show here that SUMOylated mutant ATXN7 is degraded by RNF4; this pathway mirrors that described in a study on SCA1, in which mutant ataxin-1 is modified by SUMO2 and targeted for nuclear degradation via RNF4 (Guo et al., 2014).

To summarize, we illustrated in Fig. 7B a possible mechanism for the clearance of mutant ATXN7: SUMO2 is added as poly-SUMO chains to its substrate, polyQ-ATXN7; this modification leads to the recruitment of the SUMO-targeted ubiquitin ligase RNF4 and to subsequent ubiquitination and proteasomal degradation of misfolded ATXN7.

The SUMO2/3 conjugation pathway is known to be upregulated by several protein-damaging events and is stimulated in a stress-dependent manner (Saitoh and Hinchey, 2000); for example, by nuclear accumulation of misfolded proteins. To study a correlation between SUMO2/3 and stress induced by ATXN7 aggregates, we investigated the endogenous SUMO2 protein expression both *in vitro* (Fig. 2A,B) and *in vivo* (Fig. 5). In *Atxn7^100Q/5Q^* mice, SUMO2 relocalized to ATXN7 intranuclear neuronal inclusions in cerebellar Purkinje cells and in the retinal ganglion cells, where SUMO2 expression was particularly intense. The GCL, as well as photoreceptors, were described to be degenerated in a SCA7 patient after autopsy (Gouw et al., 1994), owing to progressive accumulation of ATXN7 over time. Thus, SUMO2 colocalization with mutant ATXN7 may be linked to disease progression.

The mechanism proposed here, by which SUMO2 regulates the clearance of misfolded mutant ATXN7, makes this pathway particularly relevant to the SCA7 disease. This finding is consistent with the SUMO2/3 modification of huntingtin in HD, as SUMO2/3 is involved in the accumulation of insoluble mutant huntingtin (O'Rourke et al., 2013). Relocalization of SUMO2 into intranuclear neuronal inclusions suggests that chronic expression of polyQ-expanded ATXN7 produces a misfolded protein recognized by the SUMO pathway. Nevertheless, this response is not sufficient to cope with the accumulation of insoluble species, reflecting ongoing pathogenesis. To better understand SCA7 pathophysiology, we looked for evidence of a SUMOylation deregulation *in vivo* in the *Atxn7^100/5Q^* mouse model. Quantification of mRNA showed a decrease in the transcription of key genes – such as *Sumo1*, *Pias2*, *Pias4*, *Senp2* and *Senp6* – at 12 months, and comparison of gene expression between 6 and 12 months in *Atxn7^100/5Q^* mice showed that deregulation was stronger, especially for *Sumo1*, *Sumo2*, *Pias4* and *Senp2*, *Senp3* and *Senp6*, as the disease worsened. This downregulation of *Senp2*, *Senp3* and *Senp6*, which has paradoxical effects on SUMOylation, being involved in both the maturation of SUMO peptides as well as in de-SUMOylation, could also contribute to dysregulation of SUMOylation, particularly at later stages of the disease. Quantification of poly-SUMO2/3 proteins in the cerebellum of *Atxn7^100/5Q^* mice showed an increase in expression levels of ∼50% compared with their wild-type littermates at 12 months of age. This reveals a stabilization and longer half-life of SUMO2/3-modified proteins when the disease progresses that might be related to less-efficient proteasomal degradation. We suggest that SUMOylation contributes to the degradation of mutant ATXN7 via active proteasomes in young mice, but that degradation is compromised in aged mice, thus ATXN7 inclusions increase over time. It is well documented that proteasome activity declines during aging, and proteasomal dysfunction is associated with late-onset disorders, such as Alzheimer's disease, Parkinson's disease or HD (Saez and Vilchez, 2014).

SUMOylation was also deregulated in the R6/2 mouse model of HD: both *Sumo1* and *Sumo2* mRNA increased in the striatum at a late stage of the disease; the same pattern was observed in the mRNA expression of enzymes (*Senp1*, *Senp6*, *Pias3* and *Pias4*) involved in this pathway (O'Rourke et al., 2013).

Target specificity for SUMOylation is provided by the SUMO E3 ligases. Comparison of mRNA levels in *Atxn7^100Q/5Q^* mice and wild-type littermates showed that *Pias2* and *Pias4* mRNA expression was significantly reduced at the end of their life (12 months). Moreover, in our SCA7 cell model of overexpression, we observed that PIAS proteins (especially PIAS4), were implicated in the SUMOylation of ATXN7 (data not shown), suggesting potential target specificity.

We have shown that SUMO1 and SUMO2 colocalized with ATXN7 in the cortex of SCA7 patients (Janer et al., 2010) and in the cerebellum and retina of *Atxn7^100Q/5Q^* mice (this study). SUMO proteins and RNF4 were found to accumulate in human SCA7 cerebellar Purkinje cells, suggesting a stabilization of SUMO proteins along disease progression. The increase in cytoplasmic SUMO1 was already shown in other neurodegenerative diseases, such as multiple system atrophy (Pountney et al., 2005). Fluorescence immunohistochemistry showed that SUMO1 subdomains were frequent within and around inclusion bodies and colocalized with a lysosomal marker, cathepsin-D, in affected brain regions. This suggests that SUMO1 plays a role in lysosome function (Wong et al., 2013) and might explain the increase in cytoplasmic SUMO1 in Purkinje cells in a SCA7 brain. We have shown that the autophagy-lysosomal degradation pathway contributes to SCA7 (Alves et al., 2014). SUMO1 and the autophagy-lysosome pathway might therefore interact or intervene successively in SCA7 if the SUMO pathway and proteasome cannot handle the amount of misfolded accumulated proteins in the nucleus: this mechanism has been proposed for huntingtin degradation as well (Thompson et al., 2009). There is *in vivo* evidence that SCA1 and huntingtin mutations affect SUMOylation. In HD brains, SUMO2-modified HMW species were detected, as well as huntingtin-positive species migrating at the same level, strongly suggesting that huntingtin modification by SUMO2 is active in HD (O'Rourke et al., 2013). Similarly, in *Atxn7^100/5Q^* mice cerebella, we detected SUMO-2/3 HMW species and mutant ATXN7 in the stacking gel. Our results confirm the importance of the SUMO2 pathway and RNF4 in SCA7 and indicate that proteins involved in the SUMOylation or deSUMOylation of ATXN7 could represent potential targets for therapeutic intervention. A small molecule inducer of the SUMO-activating enzyme E1 ligase has been found in a very specific situation (Kho et al., 2015); however, most studies in the SUMO field target inhibition of this pathway, of utmost importance in the cancer field (He et al., 2017). SUMO E3 ligases may be key actors to keep the right balance between normal protein homeostasis, and deregulation of degradation pathways that lead to neuronal dysfunction and neurodegeneration. There are no pharmacological molecules to induce the PIAS proteins (even fewer to target brain). Design of viral vectors expressing PIAS proteins could be a possible strategy to induce, in brain, a high percentage of SUMOylated ATXN7. As IFN-beta acts on both upregulating PML clastosomes (Chort et al., 2013), and boosts the SUMO2/3-conjugated protein levels in cerebellum (as indicated in this study), we propose that it could be the best therapeutic agent acting on two complementary pathways to finally target and degrade mutant ATXN7 via the proteasome.

## MATERIALS AND METHODS

### Plasmids and site-directed mutagenesis

All ATXN7 constructs contain full-length ATXN7 as described (Janer et al., 2006, 2010; Zander et al., 2001). ATXN7-100Q was cloned in the Clontech EGFP-N1 vector (Zander et al., 2001). ATXN7-72Q mutant KR is mutated at position K257 identified as the major SUMO site (Janer et al., 2010). The ATXN7-72Q 7KR mutant contains seven lysines mutated into arginine at the following positions: 117, 223, 226, 257, 287, 321 and 858. FLAG-SUMO2-pSG5, HA-SUMO2-pSG5 and plasmids coding for wild-type and mutant RNF4 in the four SIMs and in the RING finger were all cloned in pSG5. The SIM mutations were those described by Tatham et al. (2008): RNF4 dSIM corresponds to mutated SIM1 (I40A, L42A and V43A), mutated SIM2 (I50A, V51A and L53A), mutated SIM3 (V61A, V62A and V63A) and mutated SIM4 (V71A, V72A, I73A and V74A). The RING finger mutation (dHC) changes His160 to Ala and Cys163 to Ser. All constructs were sequenced.

### RNA interference

Dharmacon On Target Plus SMARTpools (Thermo Fisher Scientific) were used for siRNA disruption of RNF4 (L-006557) and non-targeting siRNA (D-001810). HeLa cells were seeded in six-well dishes at a density of 1.5×10^6^ cells per well in Dulbecco's modified Eagle medium (DMEM). The next day, the cells were transfected with 75 pmol RNA in 4 μl RNAi Max (Invitrogen), according to the manufacturer’s instructions. After 24 h, the cells were transfected with 2 μg ATXN7-72Q using Lipofectamine 2000 (Invitrogen). After 48 h, the cells were harvested for preparation of whole extracts or denaturing immunoprecipitation.

### Primary antibodies

Antibodies used in western blot (WB), immunofluorescence (IF), immunohistochemical (IHC) and immunoprecipitation (IP) analyses are presented in Table S1.

### Cell culture

HeLa, HEK293 and MCF7 cells were maintained in DMEM (Invitrogen) supplemented with 10% fetal bovine serum and penicillin-streptomycin (100 UI/ml-100 µg/ml). Cell lines were purchased from American Type Culture Collection (LGC Standards, France) and tested for mycoplasma contamination. Cell lines were transfected with Lipofectamine 2000 (Invitrogen) as prescribed. Western blots were performed after 48 h of expression. HeLa cells were analyzed by immunofluorescence 40-45 h post-transfection. Depending on the analysis, cells were either scraped in PBS containing N-ethyl maleimide (NEM), centrifuged and lysed, or lysed directly in 2% SDS Laemmli sample buffer or, for immunoprecipitation, lysed in denaturing buffer containing 1% SDS.

### Cell lysate fractionation, mouse brain extracts, immunoblot and filter retardation assay

Cells were washed and harvested in PBS supplemented with 20 mM NEM, pelleted and lysed for 30 min on ice in lysis buffer containing 50 mM Tris-HCl, pH 8.8, 100 mM NaCl, 5 mM MgCl_2_, 0.5% NP-40, 1 mM EDTA, 20 mM NEM and 250 IU/ml benzonase (Merck) supplemented with a cocktail of protease inhibitors (Complete and Pefabloc, Roche). Total extracts were centrifuged at 16,000 ***g*** for 15 min at 4°C to separate soluble proteins from aggregates. Supernatants were analyzed by immunoblotting (NS fraction). Pellets were further incubated for 30 min on ice in a buffer containing 20 mM Tris-HCl, pH 8.0, 15 mM MgCl_2_, and 250 IU/ml benzonase. Mouse brain samples (cerebella, cortex, brainstem) were ground (Tissue Lyser II, Quiagen) in ice-cold RIPA lysis buffer containing 50 mM Tris-HCl, pH 8.8, 150 mM NaCl, 1 mM EDTA, 1% Na deoxycholate, 0.1% SDS, 1% NP-40, 20 mM NEM and 250 IU/ml benzonase (Merck) supplemented with protease inhibitors (Complete, Roche). Extracts were sonicated (four pulses of 10 s) and centrifuged at 16,000 ***g*** for 15 min at 4°C, and supernatants were collected for immunoblotting. Protein concentrations were determined by Bradford assay (Bio-Rad). Samples containing 25 µg (HEK293, HeLa cell extracts) or 50 µg (mouse brain extracts) of protein were resolved on pre-cast 4-12% (Invitrogen), 7.5% or 4-20% (Bio-Rad) gels, transferred onto nitrocellulose membranes (Protran, Whatman) by liquid transfer for 1.5 h, stained with Ponceau Red and blocked in 5% non-fat milk. Selected primary antibodies were incubated with the membranes overnight at 4°C (Table S1); horseradish peroxidase-conjugated or fluorescent secondary antibodies were incubated for 2 h at room temperature. Membranes were then incubated with enhanced chemiluminescence substrate (Pierce); chemiluminescence or fluorescence signals were revealed on film (ECL, Amersham Hyperfilm) or captured with an Odyssey Imaging (Li-COR) system. Densitometry was carried out using ImageJ software (NIH). The pellet was analyzed in a filter retardation assay (SR fraction): samples (40 µg) were boiled for 5 min in 2% SDS buffer and filtered on a BRL dot-blot filtration unit through a cellulose acetate membrane (Schleicher and Schuell, 0.2 µm pore size) equilibrated with 0.1% SDS (Sittler et al., 1998; Wanker et al., 1999).

### SUMO immunoprecipitation

SUMO immunoprecipitation (SUMO-IP) was performed as described (Barysch et al., 2014; Becker et al., 2013). MCF7 cells were grown to confluence in 15-cm dishes, with three plates per immunoprecipitation condition (control, SUMO1, SUMO2). Cells were washed in cold PBS with 10 mM NEM. Then, 250 μl of 2× lysis buffer (1× PBS, 2% SDS, 10 mM EDTA, 10 mM EGTA, 20 mM NEM, 2 mM Pefabloc and 2 μg/ml each of aprotinin, leupeptin and pepstatin) was added to each plate and cells were scraped. MCF7 lysates were sonicated and then boiled in 50 mM dithiothreitol (DTT), followed by a 10-fold dilution in RIPA buffer. SUMOylated ATXN7 in MCF7 lysates was immunoprecipitated with antibody-coupled beads (mouse SUMO1, clone 21C7; mouse SUMO2, clone 8A2) overnight at 4**°**C; SUMO-conjugates were eluted with an excess of epitope-spanning peptides to ensure specificity. A pre-elution step (without peptide) was followed by two elution steps (incubation with epitope-spanning peptides: SUMO1 21C7, VPMNSLRFLFE; SUMO2 8A2, IRFRFDGQPI). To concentrate endogenous SUMO-modified ATXN7, the eluted proteins were precipitated with trichloroacetic acid. To analyze potential SUMO targets, 40 μl (50%) of the precipitated eluate and 20 μl (0.2%) of the input and flow-through were loaded on 5-20% SDS-PAGE gels.

### *In vivo* SUMOylation and denaturing immunoprecipitation

HEK293 cells were transfected with HA-ATXN7-72Q, FLAG-SUMO-2 and additional constructs as indicated in the figures. At 32 h after transfection, cells were treated with 1 μM epoxomicin or DMSO for 16 h, and harvested in 100 μl IP-lysis buffer containing 50 mM Tris-HCl, pH 7.5, 150 mM NaCl, 0.5% NP-40, 0.5% Triton X-100, 10 mM NEM, supplemented with 1% SDS, 50 mM DTT, 250 IU/ml benzonase and protease inhibitors. For denaturing immunoprecipitation (d-IP), cell lysates were boiled at 95°C for 10 min. One aliquot (5%) of input was saved for western blot analysis. The remaining lysate was diluted 10-fold in IP-lysis buffer to reduce the SDS concentration. Lysates were centrifuged at 16,000 ***g*** for 15 min at 4°C. Protein concentrations were determined by BCA assay (Pierce). Lysates (800 μg) were incubated with anti-HA beads (Sigma-Aldrich, A2095) at 4°C on a rotator for 4 h or overnight. The beads were washed four times with IP-lysis buffer and boiled in 60 μl 2% SDS Laemmli buffer. Proteins eluted from beads were analyzed by immunoblotting with anti-HA and anti-SUMO2 antibodies. To compare the levels of ATXN7 SUMOylated species, d-IP products were analyzed by immmunoblotting after normalization to similar levels of unmodified ATXN7 by densitometry with ImageJ.

### Proximity ligation assay

Proximity ligation assay (Olink Bioscience) allows the detection of proteins located in close proximity (<40 nm); it is based on the *in situ* proximity ligation of DNA linked to secondary antibodies followed by PCR amplification. Primary antibodies were anti-1C1 and anti-SUMO1, anti-1C1 and anti-SUMO2/3, anti-ATXN7 and anti-T7 tag. The specificity of the PLA signal was confirmed by the lack of signal in cells treated with detection probes (PLA+ and PLA−) and one of two antibodies (anti-1C1 for ATXN7 protein, or anti-SUMO2/3 for SUMO2).

### Animals

Heterozygous *Atxn7^100Q/5Q^* knock-in mice carrying 100Q CAG repeats in the mouse *Sca7* locus on the pathological allele, kindly provided by Prof. H. Zoghbi (Baylor College of Medicine, Houston, TX, USA), were housed in a temperature-controlled room and maintained on a 12 h light/dark cycle (Chen et al., 2012). Food and water were available *ad libitum*. Immunohistochemical analyses were performed at 12 months of age (late-stage disease), compared with 12-month-old wild-type littermates. Western blot analyses from the cerebella and cortex of *Atxn7^100Q/5Q^* mice (*n*=4, 2 males, 2 females) compared with wild-type littermates (*n*=4, 2 males, 2 females) were performed at 12 months. Western blot analyses from heterozygous *SCA7^266Q/5Q^* mice cerebella (11-week-old animals; *n*=4 MSA-treated and *n*=5 IFN-beta-treated males) were performed. Quantitative RT-PCR of mRNA was performed in *Atxn7^100Q/5Q^* mice (*n*=5, 3 males, 2 females) (*n*=5, 2 males, 3 females) compared with wild-type littermates at 6 and 12 months (*n*=5 and *n*=5, respectively, 3 males, 2 females per group). For the *SCA7^266Q/5Q^* knock-in line, we used RNA extracted from the cerebella of *n*=5 MSA-treated and *n*=5 IFN-beta-treated males (heterozygous animals, 266Q/5Q), from 5 weeks of age until 11 weeks of age at sacrifice (Chort et al., 2013), to perform quantitative RT-PCR. The experiments were carried out in accordance with the European Community Council directive (86/609/EEC) for the care and use of laboratory animals and were approved by the Commission Génie Génétique of the French Ministry for Scientific Research and Education (06/26/2010).

### RNA purification and quantitative RT-PCR

Cerebella were dissected from 6- and 12-month-old *Atxn7^100Q/5Q^* mice and wild-type littermates. Tissues were homogenized with an electric pestle (Sigma-Aldrich) in QIAzol (Qiagen), and total RNA was isolated using RNeasy**^®^** Lipid Tissue Mini kit (Qiagen) including an RNase-free DNase step. The quality of total RNA was controlled (Bioanalyzer 2100, Agilent). Total RNA (1 μg) was reverse transcribed using the iScript cDNA synthesis kit (Bio-Rad) according to the manufacturer’s instructions. mRNAs were quantified by real-time RT-PCR in a Roche LightCycler LC480 sequence detection system (Roche). Oligonucleotide primer pairs were obtained from MWG Operon (France), designed with Oligo Explorer 1.0 and verified for specificity with the NCBI Blast engine (www.ncbi.nlm.nih.gov/BLAST) using the ‘nearly exact short match’ program (Table S2). The end-point PCR (35 cycles) was performed with LightCycler 480 SYBER Green I Master (Roche) on 20 ng reverse transcription product. After amplification, melting curves for the PCR products were analyzed to confirm amplification specificity. The relative levels of mRNA were standardized to *Rplp0* ribosomal RNA, and to beta-2microglobulin (*B2m*) as non-variant RNA species, according to Boda et al. (2009): forward primer for *Rplp0* 5′-GGCTGATCCATCTGCATTTGCG-3′; reverse primer for *Rplp0* 5′-ATCTGATTCCTCCGACTCTTCCTTTG-3′; forward primer for *B2m* 5′-GCTATCCAGAAAACCCCTCAA-3′; reverse primer for *B2m* 5′-CATGTCTCGATCCCAGTAGACGGT-3′. The primers of the SUMO pathway – *Pias1*, *Pias2*, *Pias3*, *Pias4*, *Senp1*, *Senp2*, *Senp3* and *Senp6* – were those published by O'Rourke et al. (2013). Each sample was quantified in triplicate. Expression levels were determined with the *2^−ΔΔCT^* algorithm following Applied Biosystem guidelines and Livak and Schmittgen (2001).

### Immunofluorescence and confocal microscopy

HeLa cells were plated on poly-lysine (Sigma-Aldrich)-coated glass coverslips. At 40-45 h post-transfection, cells were fixed for 20 min with 4% paraformaldehyde at room temperature, washed with PBS, permeabilized in 0.1% Triton X-100 in PBS for 15 min, washed twice in PBS, and blocked in 4% bovine serum albumin (BSA) in PBS for 1 h. Primary antibodies (Table S1) were diluted in 4% BSA in PBS and incubated overnight at 4°C. The cells were then washed three times in PBS, and secondary antibodies, diluted in 4% BSA in PBS, were applied for 2 h at room temperature and washed. Secondary antibodies were Alexa Fluor^®^ 488-conjugated goat anti-mouse or goat anti-rabbit IgG (Invitrogen), Alexa Fluor^®^ 568-conjugated goat anti-mouse or goat anti-rabbit IgG (Invitrogen) and Fluoprobes^®^ 647-conjugated donkey anti-rabbit (Interchim), used at 1:500. The cells were then incubated with 4′,6-diamidino-2-phenylindole (DAPI) diluted in PBS for 15 min, washed in PBS and mounted with Fluorescent Mounting Medium (Dako). Confocal images were acquired at room temperature with an Olympus BX 61 microscope equipped with ×60/1.35 lens and a Fluoview FV-1000 image acquisition system. For Fig. 2B, confocal images were acquired with a Leica SP5 inverted microscope equipped with a ×63 lens and a Leica LAS image acquisition system. Images were analyzed using ImageJ and the colocalization plugin JACoP (Just Another Colocalization Plugin), as described in Bolte and Cordelières (2006).

### Human postmortem brain

Formalin-fixed, paraffin-embedded cerebellar tissue from two SCA7 patients with morphologically and genetically confirmed SCA7 (57 and 10 years old at death; with 47 CAG repeats and 85 CAG repeats on the mutant allele, respectively), and a control subject with no evidence of neurological disease (52 years old at death) were obtained from the Brain Bank of the Groningen Medical Center (Groningen, The Netherlands) and from the Department of Neuropathology of the Pitié-Salpêtrière Hospital (Paris, France). All experiments with human tissue were conducted under the ‘Code of Conduct for dealing responsibly with human tissue in the context of health research’ published by the Federation of Dutch Medical Scientific Societies in 2011. The study was conducted in accordance with the Declaration of Helsinki and all tissue donor patients provided written informed consent.

### Immunohistochemical analysis

Mouse brain: mice received an overdose of sodium pentobarbital and were perfused transcardially with 4% paraformaldehyde in 0.1 M PBS. The brains were post-fixed in 4% paraformaldehyde for 24 h and cryoprotected in 30% sucrose-PBS for 48 h at 4°C. Immunostaining was performed on 20-μm-thick frozen cryostat sections. Antigen retrieval, permeabilization and blocking were performed as for human samples (see below). Sections were incubated for 48 h at 4°C with primary antibodies (Table S1) followed by the appropriate Alexa Fluor^®^ dye-conjugated secondary antibodies (Invitrogen, 1:500): Alexa Fluor^®^ 488-conjugated goat anti-rabbit (ATXN7) and Alexa Fluor^®^ 568-conjugated goat anti-mouse (SUMO2, clone 8A2). Sections were incubated with DAPI, washed and mounted with Fluorescence Mounting Medium (S3023, Dako).

Human brain: 5-μm sections were cut from paraffin-embedded brain samples and stained with anti-ATXN7, anti-SUMO1, anti-SUMO2 and anti-RNF4 antibodies. Antigens were retrieved by boiling the sections in 1 mM citrate buffer, pH 6.0, in a microwave oven at 350 W. Sections were permeabilized with PBS and 0.1% Triton X-100 and blocked by incubation for 2 h at room temperature in PBS containing 3% BSA, 4% normal goat serum, 0.1% Triton X-100. Sections were incubated for 48 h at 4°C with primary antibodies (Table S1). Secondary antibodies for 3,3′-diaminobenzidine (DAB) staining were biotinylated goat anti-rabbit or goat anti-mouse antibodies (BA-9200, BA-1000, respectively; Vector Laboratories) at 1:250. DAB staining was carried out with the Vectastain ABC kit (Pierce). Images of immunostained sections were acquired with LAS V3.8 (Leica) software, at room temperature, on a bright-field Leica DM 4000B microscope equipped with a ×100/1.35 lens and a Leica DFC500 digital camera.

### Statistical analysis

For statistical analysis, we performed either two-tailed Student's *t*-tests (to analyze imaging and biochemical data) or one-way ANOVA (to analyze mRNA expression by quantitative RT-PCR) with Prism6 (GraphPad) and with R software (https://www.r-project.org/); *P*<0.05 was considered statistically significant.

## Supplementary Material

Supplementary information
