# Supplementary material for: SUMOylation by SUMO2 is implicated in the degradation of misfolded ataxin-7 via RNF4 in SCA7 models
Source: Dis Model Mech. 2019 Jan 11;12(1):dmm036145. doi: 10.1242/dmm.036145 (PMC6361149; doi:10.1242/dmm.036145)
Supplement: Supplementary information [file dmm-12-036145-s1.pdf]

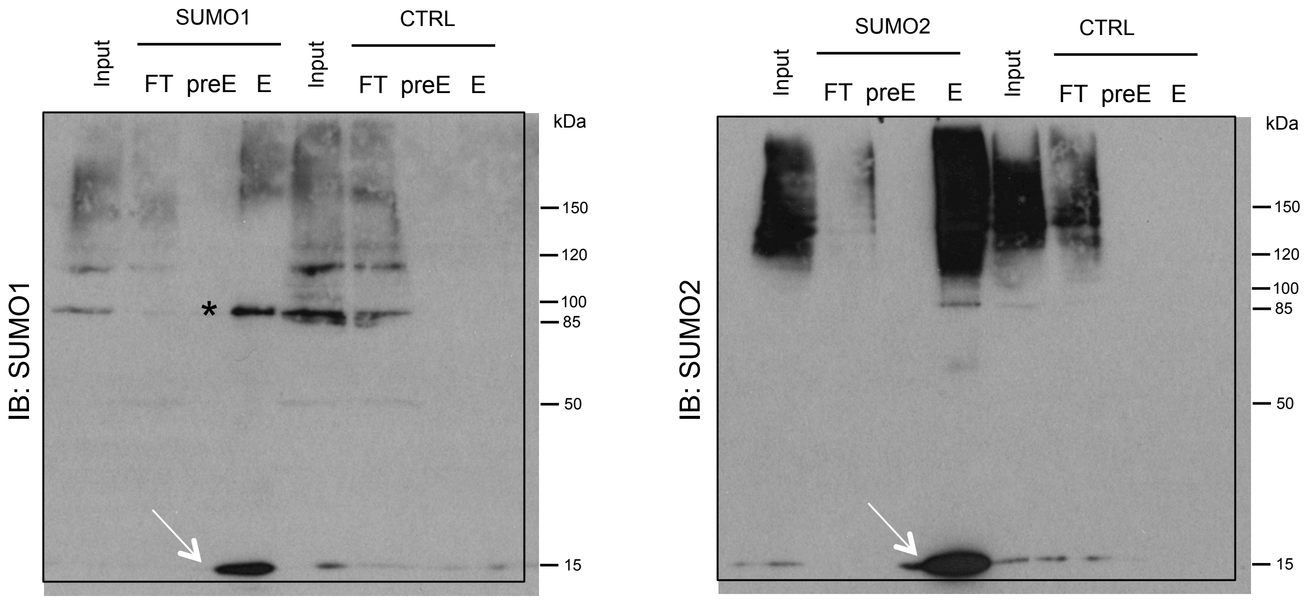

Figure S1. ATXN7 SUMOylation by SUMO-immunoprecipitation : Controls for SUMO1 and SUMO2 IP

A) MCF7 cell lysate was subjected to denaturing immunoprecipitations with beads coupled to monoclonal antibodies against SUMO1, SUMO2 or IgG (control) (see Fig.1A). IP efficiency in controls shown on immunoblots revealed with anti-SUMO1 and anti-SUMO2 antibodies: eluted free SUMO1 and SUMO2 are indicated by white arrows. FT, flow-through; preE, pre-elution wash; E, eluate. *SUMOylated RanGAP1.

Disease Models & Mechanisms • Supplementary information

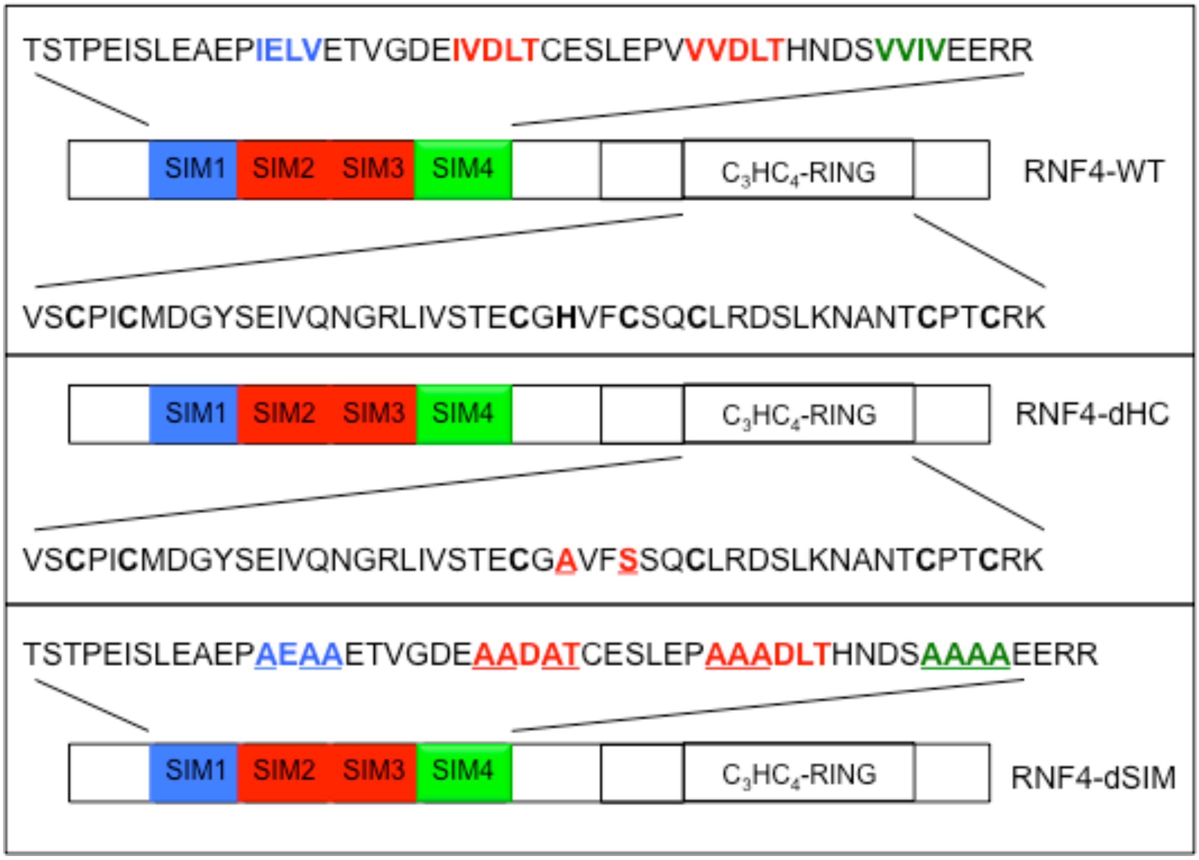

Figure S2. Schematic representation of RNF4 WT and the RNF4 mutants dHC and dSIM
SIM domains are indicated, the mutations are underlined. The RING and the mutations are underlined.

Disease Models & Mechanisms • Supplementary information

Table S1. Primary antibodies used in western blot (WB), immunofluorescence (IF), immunohistochemical (IHC) and immunoprecipitation (IP) analyses.

Primary Antibodies	Species	Source	Catalog number	WB	IHC	IF	IP
Ataxin-7	Mouse	Yvert et al., 2001	1C1			1:1000	
Ataxin-7	Rabbit	Thermo Scientific	PA1-749	1:2000	1:1000	1:1000	2,5 ug/mg of protein
FLAG tag	Mouse	Sigma	M2 F3165	1:500			
HA tag	Mouse	Covance	MMS-101P	1:2000			
PML H-238	Rabbit	Santa Cruz	Sc-5621	1 :2000		1:1000	
PML clone 36.1	Mouse	Millipore	MAB3738	1:2000			
PML	Chicken	Lallemand et al. 2008				1:2000	
RanGAP1	Goat	Barysch et al., 2014		1:2000			
RNF4	Rabbit	Hakli et al., 2005		1:1000	1:200	1:500	
SUMO-1	Rabbit	Abcam	ab11672	1:2000	1:500	1:500	
SUMO-1 21C7 (coupled-beads)	Mouse	Barysch et al., 2014	21C7				10 ul/mg of protein
SUMO-2 8A2 (coupled-beads)	Mouse	Barysch et al., 2014	8A2				10 ul/mg of protein
SUMO-2 clone 8A2	Mouse	Barysch et al., 2014	8A2	1:1000	1:250	1:500	
SUMO-2/3	Rabbit	Abcam	ab3742	1:2000	1:500	1:500	
Ubiquitin	Mouse	Santa Cruz	Sc-8017	1:2000			
Multiubiquitin Chain clone FK2	Mouse	Biomol	Cay14220	1:5000		1:500	
α-Tubulin	Rabbit	Abcam	ab4074	1:2000			

Disease Models & Mechanisms • Supplementary information

Table S2. Primer sequences.

qRT-PCR Primers	Sequence
Pias1-F	5'-GTCTCCTACGTCACCACTAAG-3'
Pias1-R	5'-GCATAGGCGTCATGTGGAAG-3'
Pias2-F	5'-CCGTGCAGTGACTTGTACAC-3'
Pias2-R	5'-CTCATCCACATCAGAACAGTC-3'
Pias3-F	5'-GGTGAGGCAATTGACTGCAG-3'
Pias3-R	5'-GTAGTAGCCACTTCACTGTCG-3'
Pias4-F	5'-GACGCTAGTGGCCAAGATGG-3'
Pias4-R	5'-GTCACCAGTTCGTGCTTCAG-3'
Senp1-F	5'-GCTGACTGACAGTGAAGACG-3'
Senp1-R	5'-GAGTCTGAATGTCTTTGCGTG-3'
Senp2-F	5'-GAAGGTGCTCATGGAAGCACG-3'
Senp2-R	5'-CTGACGCCATGATTCTGCTC-3'
Senp3-F	5'-CAGGCAACGCTATGGTAAGG-3'
Senp3-R	5'-CTGTGTCCATGACCAGATCTC-3'
Senp6-F	5'-CCTCCTTATGGACTCACTCAG-3'
Senp6-R	5'-CTACATACTGCAGCACATAGAC-3'
Sumo1-F	5'-GTGAATCCACGTCACCATGTC-3'
Sumo1-R	5'-GTATCTCACTGCTATCCTGTC-3'
Sumo2-F	5'-GTCAATGAGGCAGATCAGATTC-3'
Sumo2-R	5'-CACATCAATCGTATCTTCATCC-3'
Rplp0-F	5'-GGCTGATCCATCTGCATTTGCG-3'
Rplp0-R	5'-ATCTGATTCCTCCGACTCTTCCTTTG-3'
B2m-F	5'-GTCGTGCTTGCCATTCAGA-3'
B2m-R	5'-CATGTCTCGATCCCAGTAGACGGT-3'

Disease Models & Mechanisms • Supplementary information
